# The pyroptosis mediated biomarker pattern: an emerging diagnostic approach for Parkinson’s disease

**DOI:** 10.1186/s11658-023-00516-y

**Published:** 2024-01-03

**Authors:** Junhan Liang, Zhirong Wan, Cheng Qian, Madiha Rasheed, Changling Cao, Jingyan Sun, Xuezhe Wang, Zixuan Chen, Yulin Deng

**Affiliations:** 1 Beijing Key Laboratory for Separation and Analysis in Biomedicine and Pharmaceuticals, School of Medical Technology, Beijing Institute of Technology, Zhongguancun South Street, Haidian District, Beijing, 100081 People’s Republic of China; 2 Department of Neurology, Aerospace Center Hospital, Beijing, 100049 People’s Republic of China; 3 School of Life Sciences, Beijing Institute of Technology, Beijing, 100081 People’s Republic of China

**Keywords:** Parkinson’s disease, Biomarker patterns, Machine learning

## Abstract

**Background:**

Parkinson’s disease (PD) affects 1% of people over 60, and long-term levodopa treatment can cause side effects. Early diagnosis is of great significance in slowing down the pathological process of PD. Multiple pieces of evidence showed that non-coding RNAs (ncRNAs) could participate in the progression of PD pathology. Pyroptosis is known to be regulated by ncRNAs as a key pathological feature of PD. Therefore, evaluating ncRNAs and pyroptosis-related proteins in serum could be worthy biomarkers for early diagnosis of PD.

**Methods:**

NcRNAs and pyroptosis/inflammation mRNA levels were measured with reverse transcriptase quantitative polymerase chain reaction (RT-qPCR). Luciferase assays were performed to confirm GSDME as a target of miR-675-5p and HMGB1 as a target of miR-1247-5p. In the serum of healthy controls (*n* = 106) and PD patients (*n* = 104), RT-qPCR was utilized to assess miR-675-5p, miR-1247-5p, and two related ncRNAs (circSLC8A1and lncH19) levels. The enzyme-linked immunosorbent assay measured serum levels of pyroptosis-related proteins in controls (*n* = 54) and PD patients (*n* = 70).

**Results:**

Our data demonstrated that miR-675-5p and miR-1247-5p significantly changed in PD neuron and animal models. Overexpressed miR-675-5p or downregulated miR-1247-5p could regulate pyroptosis and inflammation in PD neuron models. Using the random forest algorithm, we constructed a classifier based on PD neuron-pyroptosis pathology (four ncRNAs and six proteins) having better predictive power than single biomarkers (AUC = 92%). Additionally, we verified the performance of the classifier in early-stage PD patients (AUC ≥ 88%).

**Conclusion:**

Serum pyroptosis-related ncRNAs and proteins could serve as reliable, inexpensive, and non-invasive diagnostic biomarkers for PD.

**Limitations:**

All participants were from the same region. Additionally, longitudinal studies in the aged population are required to explore the practical application value of the classifier.

**Supplementary Information:**

The online version contains supplementary material available at 10.1186/s11658-023-00516-y.

## Introduction

Parkinson’s disease is the second most common neurodegenerative disorder after Alzheimer's disease, affecting 1% of the population over 60 years [1]. Clinical studies have revealed motor deficits in PD patients, like resting tremors, muscle rigidity, and bradykinesia [2–5], appearing after the loss of 50–70% of dopaminergic neurons in the substantia nigra pars compacta (SNpc) and Lewy bodies formation [6, 7]. Unfortunately, the late onset of these pathological symptoms makes PD a complex neurological condition with several clinical challenges, including the inability to diagnose the disease in its early stages. Additionally, the practical applications of modern diagnostic techniques for PD diagnosis based on clinical signs and symptoms are still insufficiently precise and accurate, leading to misdiagnosis due to the clinical overlap between neurodegenerative diseases and causing severe complications onwards [4, 8]. For instance, medical imaging, which is an auxiliary technique for PD diagnosis, is still not widely acknowledged by the general public due to its high cost and time consumption. Moreover, it has been argued that these measurements may be influenced by homeostatic regulatory mechanisms, particularly in the early disease stages, making it challenging to utilize them as precise indicators of prodromal PD [5, 9–11]. As a result, PD diagnosis in patients and the precise determination of disease progression has become a major concern.

Recently, biomarkers from serum, cerebrospinal fluid (CSF), and saliva have gained momentum as a possible invasive tool for diagnosing PD. It is proposed that a valid and precise diagnostic biomarker can recognize PD in its earliest stages and may distinguish it from other neurodegenerative illnesses before clinical motor symptoms appear [10, 12]. Until now, numerous studies have reported the existence of biomarkers in human body fluids such as Apolipoprotein A1 in the serum [13], α-synuclein(α-syn) aggregation in the CSF [14], central nervous system (CNS)-derived extracellular vesicles [15] and SNCA intron-1 methylation in the blood [16]. However, the diagnostic accuracy of these previously reported biomarkers remained unsatisfactory, with plasma EV α-syn demonstrating only 65.4% accuracy [17]. Henceforth, it highlights the necessity of investigating precise biomarkers for early-stage PD diagnosis.

MicroRNAs (miRNAs), as one of the epigenetic factors, have been known to play a crucial role in PD development; for instance, miR-7, miR-124 and miR-29 regulate key pathogenic signaling pathways in PD [18–20], where their dysregulated expression is influenced by pathogenic and other factors [21]. Nevertheless, the unique properties of miRNAs, such as high stability, tissue enrichment, sample quantification tests, etc. suggest them as potential biomarkers for PD. Accumulating evidence has shown that miRNAs significantly influence the degenerative process of PD through their cross-talk with circular RNAs (circRNAs) and long non-coding RNAs (lncRNAs) by regulating key pathological pathways [22, 23]. Since PD patients have undergone a variety of pharmaceutical therapy, motor rehabilitation, and dietary restrictions in terms of quantity and quality [24–26], the validity of miRNAs as biomarkers can now be taken into consideration. Additionally, combining them with other types of biomarkers may also increase the sensitivity and precision of PD diagnosis [27]. miR-675-5p and miR-1247-5p are reported to play evident roles in many cancer and neurodegenerative diseases by regulating targets to impact various vital cellular processes, signifying their therapeutic and diagnostic potential for various neurological malignancies [28–30]. Recent studies have demonstrated that excessive Mn exposure to neuronal cells has lowered the expressions of miR-675-5p by sponging with lncSh2d3c, resulting in the neuronal apoptosis through lncSh2d3c/mmu-miR-675–5p/Chmp4b/Bax axis and aggravated neurodegenerative processes [31] However, in glioma studies, overexpressed miR-675-5p has promoted glioma cell proliferation migration and invasion by downregulating various tumour suppressor genes, such as retinoblastoma 1(RB1) [32]. Whereas knocking down miR-675-5p resulted in the upregulated RB1 gene following reduced tumor growth [32]. Similarly, miR-1247-5p displayed tumor suppressor response in human astroglioma U251 cells via the miR-1247-5p/CDC14B/p53 signaling axis that triggered apoptosis and inhibited tumor cell proliferation [30, 33, 34]. Nevertheless, even though the miR-675-5p and miR-1247-5p play a significant role in several neurological processes, it is still unclear how miR-675-5p and miR-1247-5p may deregulate neuronal processes and contribute Parkinson’s development.

Dopaminergic neuron (DAN) loss is a clinical hallmark which is proportional to the degenerative process of PD, triggered by NLRP3 inflammasome activation in microglia and results in neuroinflammation [35]. Convincing studies have shown that neuroinflammation occurs at the prodromal stage of PD, even earlier than motor symptoms [10, 36], supporting that neuroinflammation-related biomarkers involved in the DANs loss can serve as potential early diagnostic biomarkers of PD. Pyroptosis, an inflammatory programmed cell death, has been associated with the onset and progression of neurodegenerative illnesses like PD [22, 37]. Converging evidence has shown that NLRP3 inflammasomes are activated by Gasdermin D (GSDMD), causing DANs to undergo pyroptosis in PD mice and MPTP or MPP^+^ cell models [38, 39]. GSDMD belongs to the Gasdermin family, containing Gasdermin A, Gasdermin B, Gasdermin C, and DFNA5/Gasdermin E(GSDME). Among them, GSDME is proposed to act as a dual switch function between apoptosis and pyroptosis, controlled by dynamic caspase-3 variation [40, 41]. GSDME causes cell pyroptosis by inserting its cleaved GSDME-N terminal (GSDME-N) into the cell membrane [42] and induces apoptosis by permeabilizing the mitochondrial membrane and promoting the release of cytochrome c. During cellular damage, GSDME is reported to activate HMGB1 which functions as DAMP and accelerates inflammatory processes [43]. Additionally, recent studies on patients with anti-N-methyl-D-aspartate receptor (NMDAR) encephalitis have shown that serum Gasdermin proteins may be used as markers of CNS disease and are linked to the pathological process of CNS diseases [40, 42, 43]. Recent studies have shown that GSDMD activates neuroinflammation in PD [41] and inhibiting inflammasome has prevented α-syn pathology and dopaminergic neurodegeneration in mice [35]. However, it is still unclear how these pyroptosis proteins contribute to the neuronal degeneration process in PD. Our bioinformatic analysis showed that miR-675-5p targets GSDME and miR-1247-5p targets HMGB1, and as per our knowledge, (i) the interplay of miR-675-5p and miR-1247-5p in pyroptosis mediated-pathways in promoting PD pathogenesis has not been elucidated yet, particularly whether dysregulated expression of miR-675-5p and miR-1247-5p promotes PD development or expressed as an adaptive mechanism in response to neuronal damage in PD; and (ii) Additionally, how far multi-biomarker approach containing miR-675-5p and miR-1247-5p linking ncRNAs (circSLC8A1 and lncH19) and pyroptosis-related proteins such as (HMGB1, GSDME, GSDME-N, cleaved-caspase3, il-1β and il-18) can be utilized as a diagnostic pattern for evaluating PD progression. Therefore, to address these queries, we established PD models containing NM-salsolinol (NM-Sal) and aggravated α-syn levels using in-vivo and in-vitro studies. Although MPTP, a neurotoxin, is commonly used to develop various PD models [44]. Here in this study, we utilized NM-Sal, an MPTP-like endogenous neurotoxin found in elevated levels in the nigro-striatum of PD patients to establish PD in-vivo and in-vitro models according to our team’s previous studies [45–47]. Through in-vivo and invitro models, we probe the differential expression of miR-675-5p and miR-1247-5p linking ncRNAs (circSLC8A1 and lncH19) and their association with pyroptosis-mediated pathways in PD pathogenesis. Further, to identify the diagnostic potential of these ncRNAs for PD pathogenesis, we recruited the expression pattern of these miR-675-5p and miR-1247-5p linking ncRNAs (circSLC8A1 and lncH19) from the serum samples of PD patients and devised a diagnostic pattern for PD using multi-biomarker approach. Taken together, this study's novelty and potential translational impact lie in the promise of early PD diagnosis and its potential to guide the development of targeted therapeutics, making it beneficial for a wide range of researchers, clinicians, and stakeholders involved in PD research and patient care.

## Materials and methods

### CCK-8 assay

Cell viability of SH gfp and SH αsyn cells was assessed using the CCK-8 kit (Solarbio, China; Cat. No. CA1210) at different NMsal doses 0, 100, 200, 300, and 400 μM (see Additional file 4). Briefly, 1000 cells were grown in a medium of a 96-well plate in accordance with the manufacturer’s standard operating procedures. 100 μl of working solution was prepared by adding 10 μl of CCK-8 reagent with 90 μl of DMEM-F12 and added in each well, following 1 h incubation. The experimental procedure was performed in three replicates.

### PD model construction

SH-SY5Y cells (Pricella, China; Cat NO. CL-0208), including overexpressed α-syn protein (SH αsyn) and control groups (SH gfp) were prepared according to the Duan’s method [46]. Cells were cultured in BASIC DMEM/F-12 medium (Gibco,USA;Cat NO. C11330500BT) supplemented with 10% heat inactivated fetal bovine serum (Vistech,China; Cat NO.SE100-B), 100 unit/mL penicillin G sodium, and 100 μg/mL streptomycin sulfate at 37 ℃ in humidified air with 5% CO_2_. After 24 h, the medium was replaced with fresh medium containing the endogenous neurotoxin 200 μM NM-Sal to construct two different PD models: preliminary PD cell model (SH gfp + NM-Sal) and complete PD cell model (SH αsyn + NM-Sal).

The animal study was approved by the Ethics Committee of the Beijing Institute of Technology (Beijing, China, Permit #2019–0010-R/M-2020038). Male Wistar rats (Vital River, China) having overexpressed α-syn protein were prepared according to our previous work [45]. Briefly, after 12 weeks, α-syn overexpressed rats were deeply anesthetized by pentobarbital sodium at 50 mg/kg i.p and 5 μl NM-Sal(100 nM) solution was injected into the substantia nigra pars compacta (anteroposterior − 5.3 mm from bregma, mediolateral + 2.2 mm from midline, and dorsoventral − 7.7 mm below dura). Following 4 weeks, the rats were anesthetized by pentobarbital sodium 50 mg/kg and then euthanized. After perfusion with precooled saline solution, the striatum was stored at − 80 °C for further experiments.

### Transfection

SH-gfp and SH-αsyn cells were seeded onto 24-well plates at 5 × 10^4^ per well. The following day, cells were transfected with miR-675-5p mimics/miR-1247-5p inhibitor (Sangon, China) according to manufacturer’s instructions (Omiget, China; Cat No Omc-02) (see Additional file 6). Fresh media and 200 µM NM-Sal, were applied to the cells six hours after transfection. After 48 h treatment, cells were the collected for analysis.

### Quantification of ncRNA and mRNA expression levels

MiRNAs, ncRNAs and mRNAs were extracted from PD model (in-vivo, in-vitro models) and serum samples of the PD patients through serum/plasma microRNA extraction kit (GeneBetter, China; Cat No.R416) or Trizol reagent (Invitrogen, USA; Cat NO.15596018) respectively according to the manufacturer’s protocol. After analyzing the purity and concentration of RNA extracts, miRNA cDNA synthesis was performed using Script III miRNA First Stand Synthesis Kit (GeneBetter, China; Cat No. P418-25), circRNA,lncRNA and mRNA’s cDNA synthesis was performed using universal RT-PCR Kit(M-MLV) (Solarbio, China; Cat NO.RP1100). TB Green Premix Ex Taq (Takara, Japan; Cat No RR420a) was used to detect ncRNAs and mRNAs levels for qPCR quantification according to the manufacturer’s instructions. The details of all primers (Sangon, China) are mentioned in Additional file 1. After normalizing with U6/GAPDH levels, the delta-delta cycle threshold value (2^-ΔΔCt^) method was used to quantify the difference in ncRNAs’ and mRNAs’ expression between groups in comparison to the control group.

### Luciferase reporter assay

According to the miRwalk database, miR-675-5p binds to GSDME, and miR-1247-5p binds to HMGB1.To determine the binding efficiency between miR-675-5p and GSDME, miR-1247-5p and HMGB1, SH-SY5Y cells were co-transfected with miR-675-5p/miR-1247-5p mimics or mimics-NC (Sangon, China) with a luciferase construct containing GSDME/HMGB1 having wild-type binding site (Tsingke, China). Luciferase activities were analyzed using the Firefly Luciferase Reporter Gene Assay Cell Lysis Buffer (Beyotime, China; Cat No RG126S) and Dual Luciferase kit (Beyotime, China; Cat No RG088S) according to the manufacturer’s instructions.

### Study design and population

This study was approved by the Research Ethics Committee of the Aerospace Center Hospital (Beijing, China, Permit 2021-ASCH-009). A total of 210 participants (104 PD patients and 106 controls), enrolled between April 2021 and February 2022, were recruited from Aerospace center hospital. Patients with PD were diagnosed according to the UK Parkinson’s Disease Society Brain Bank diagnostic criteria [48]. Healthy controls were selected from the patient’s relatives and friends. All participants provided written informed consent for this study.

### Clinical and demographic data

Demographics and questionnaires (designed by our research team and approved by the Aerospace Center Hospital Research Ethics Committee) were obtained from all participants. The H&Y scale was used to categorize PD patient’s stages during diagnosis. PD patients falling under H&Y stage ≤ 2 was defined as early-stage patients, while PD patients falling under H&Y stage > 2 were described as “intermediate and end-stage patients”. A neurological physician recorded the drug use of PD patients to calculate levodopa equivalent daily dose (LEDD).

### Serum collection and processing

Serum samples were recruited by centrifuging PD blood samples at 3000 rpm at 4 ℃ for 20 min. Serum samples were then preserved at − 80 ℃ for further experimentation. Repeat freeze–thaw cycle was avoided throughout the whole experiment.

### Enzyme-linked immunosorbent assay (ELISA)

The serum protein levels of each participant were analyzed through the sandwich ELISA method. The ELISA kits for HMGB1 (Meike, China; Cat No.mk3547a), IL-1β (Meike, China; Cat No.mk0179a), IL-18 (Meike, China; Cat No.mk0139a), GSDME (Meike, China; Cat No.mk4194a), GSDME-N (Meike, China; Cat No.mk4190a), cleaved caspase3 (Meike, China; Cat No.mk4187a) were utilized according to the manufacturer’s instructions.

### Statistical analysis

Two-tailed student *t*-test, ANOVA and Pearson correlation analysis were used. ANOVA was used to assess the difference in serum ncRNA levels among H&Y stages relative to the control cohort. Non-normally distributed data were analyzed using the Mann–Whitney *U* or Kruskal–Wallis test. The Pearson correlation was used to assess whether LEDD, disease duration, or protein were associated with serum ncRNAs levels. All analyses were performed with GraphPad Prism (GraphPad Prism 7.04; Bethesda, MD, USA). Values within the range of *p* < 0.05 were considered statistically significant.

To build and test an optimal classifier using the randomly selected cohort (*n* = 124), miR-675-5p, miR-1247-5p, circSLC8A1, lncH19, HMGB1, il-1β, il-18, GSDME, GSDME-N, cleaved caspase3 were tested in different combinations and sensitivity, specificity and AUC values were used to estimate. We compared two statistical methods: logistic regression and random forest. Random forest was chosen due to its ability to yield an AUC of 1 in the training data set. Meanwhile, compared to logistic regression, random forest could generate a high-performance classifier by grouping several weak classifiers. Furthermore, the data set was split into training and testing sets to train and assess the classifier’s performance. The above analysis was done in the SciKit-learn machine learning library (Python 3.8).

## Results

### MiRNAs expression in PD models

Previous research has shown that the neurotoxin NM-Sal specifically damages dopaminergic neurons [49, 50]. One characteristic pathogenic aspect of PD is the abnormal aggregation of α-syn [51]. Therefore, it is speculated that PD models that incorporate both pathogenic variables are better able to mimic the diseased process.

**Fig. 1:**
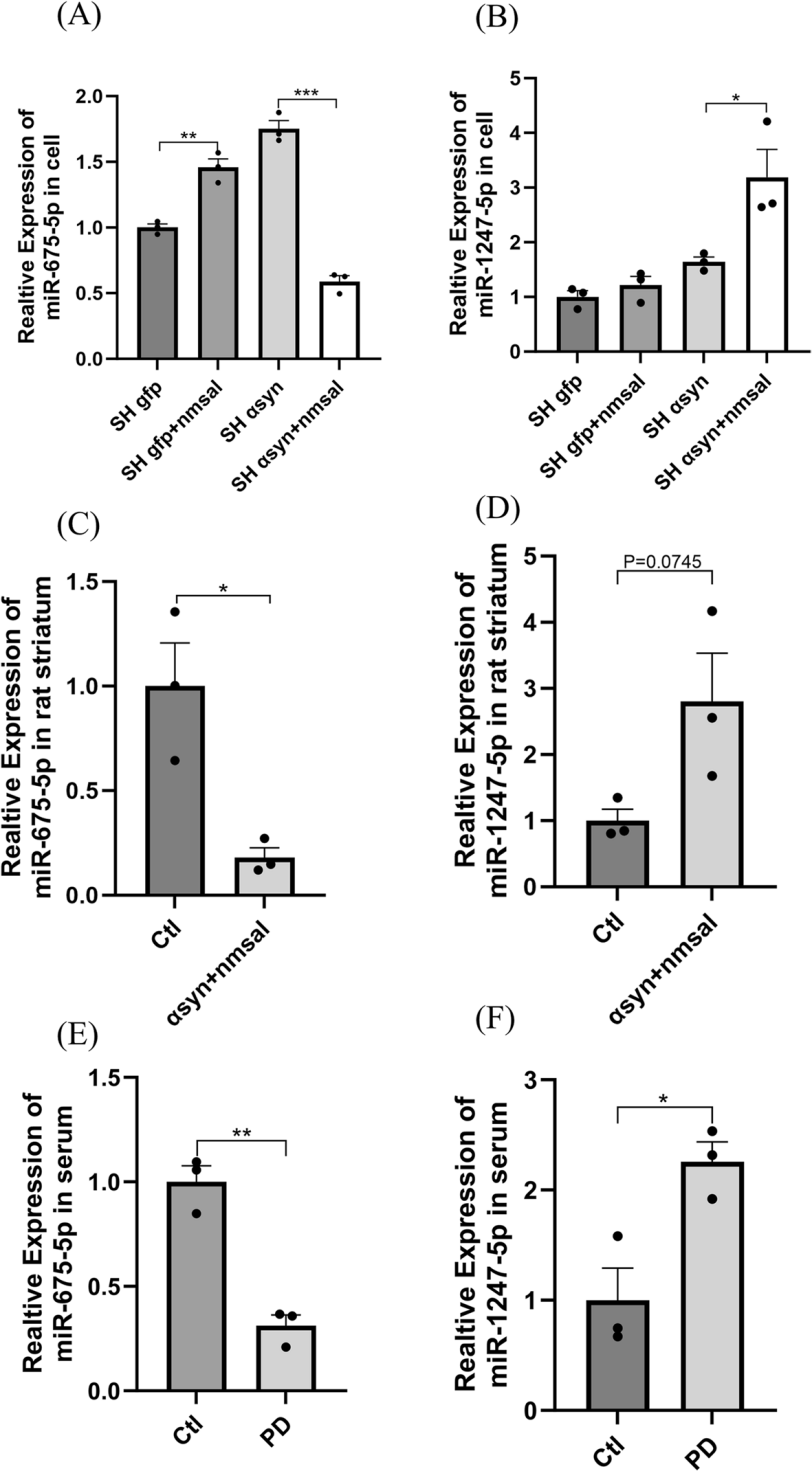
miRNA levels in PD models. The expression levels of miR-675-5p (**A**, **C** and **E**) and miR-1247-5p (**B**, **D** and **F**) were measured by RT-qPCR. The delta-delta cycle threshold value (2^-ΔΔCt^) method was used to analyze the results relative to U6. The results were compared with control groups. Data are represented as the mean ± SEM of three biological replicates, performed using three technical replicates. (Ctl, control; SH, SH-SY5Y cell;α syn, α-synuclein; NM-Sal, NM-Salsolinol; miR, microRNA;*P < 0.05; **P < 0.01;***P < 0.001)

Based on our previous work, miR-675-5p and miR-1247-5p were selected as possible biomarkers of PD. The results of PD cell and rat model both showed remarkable changes in the miR-675-5p and miR-1247-5p levels. When NM-Sal was used alone, the expression level of miR-675-5p increased significantly. However, miR-675-5p decreased significantly under two pathogenic factors (NM-Sal and α-syn) of PD (*P* = 0.0031 and *P* = 0.0001 respectively, Fig. 1A). Meanwhile, level of miR-1247-5p increased significantly under NM-Sal and α-syn levels (*P* = 0.0406, Fig. 1B). Additionally, the striatum of PD animal models also showed significantly lower expression of miR-675-5p (*P* = 0.0176, Fig. 1C), while higher expression of miR-1247-5p was observed (*P* = 0.0745, Fig. 1D). In addition, we also performed preliminary experiment in the serum samples obtained from 3 PD patients and 3 healthy controls, the expression levels of miR-675-5p (*P* = 0.0017, Fig. 1E) and miR-1247-5p (*P* = 0.0216, Fig. 1F) remained consistent with above results.

### MiR-675-5p and miR-1247-5p regulate pyroptosis and neuroinflammation in PD cell model

**Fig. 2:**
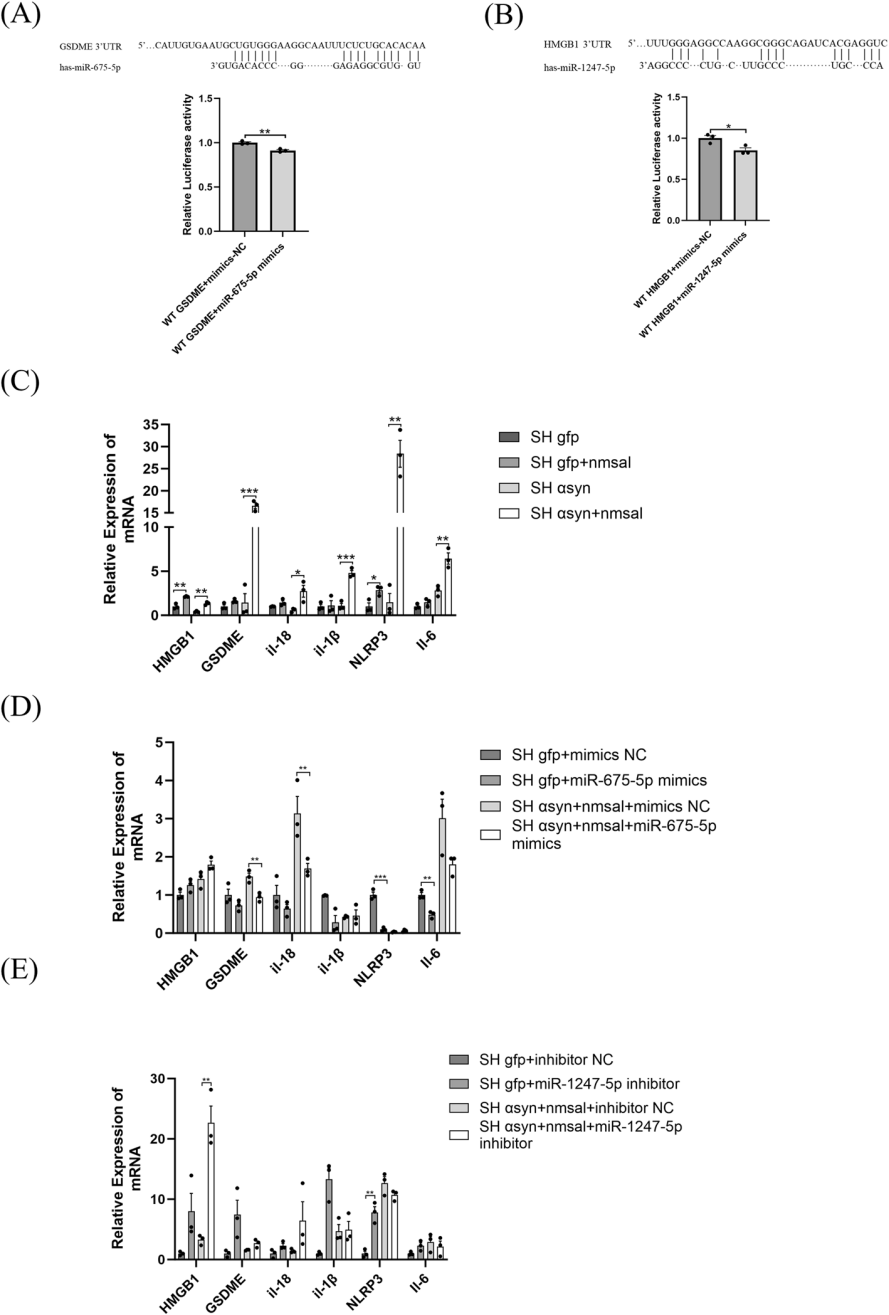
Effects of miR-675-5p/miR-1247-5p on pyroptosis/neuroinflammation in PD models. The putative miR-675-5p binding site in the 3′ UTR of GSDME and relative luciferase activity was determined by luciferase reporter assay in SH-SY5Y cells co-transfected with miR-675-5p mimics/mimics-NC and wild reporter vectors (GSDME WT) (**A**). The putative miR-1247-5p binding site in the 3′ UTR of HMGB1 and relative luciferase activity was determined by luciferase reporter assay in SH-SY5Y cells co-transfected with miR-1247-5p mimics/mimics-NC and wild reporter vectors (HMGB1 WT) (**B**). The expression levels of HMGB1, GSDME, il-18, il-1β, NLRP3 and il-6 were measured by RT-qPCR. The delta-delta cycle threshold value (2^-ΔΔCt^) method was used to analyze the results relative to GAPDH. The results were compared with control groups. Significant differences were assessed using a two-tailed student t-test. Data are represented as the mean ± SEM of three biological replicates performed using three technical replicates. (Ctl, control; SH, SH-SY5Y cell; α syn, α-synuclein; NM-Sal, NM-Salsolinol; miR, microRNA;*P < 0.05; **P < 0.01;***P < 0.001)

Using bioinformatics tools, we found that miR-675-5p had binding sites with GSDME, and miR-1247-5p had binding sites with HMGB1. It is proposed that two miRNAs (miR-675-5p and miR-1247-5p) might be involved in pyroptosis-related pathways. To confirm the speculation, the assay of dual luciferase reporter was performed in SH-SY5Y cells co-transfected with GSDME-WT or HMGB1-WT. MiR-675-5p overexpression dramatically lowered the luciferase activity of GSDME-WT vector (*P* = 0.0035, Fig. 2A). Similarly, miR-1247-5p overexpression remarkedly decreased the luciferase activity of HMGB1-WT reporter vector (*P* = 0.0303, Fig. 2B).

The functional effect of miR-675-5p and miR-1247-5p on NM-Sal-induced PD cell models was investigated. We first determined the pyroptosis and neuroinflammation related factor by RT-qPCR after treating with NM-Sal. As expected, NM-Sal led to significant pyroptosis and neuroinflammation by presenting significantly increased expression of HMGB1 (*P* = 0.0030 and *P* = 0.0012 respectively, Fig. 2C) and NLRP3 (*P* = 0.0233 and *P* = 0.0011 respectively, Fig. 2C) in both SH-gfp and SH-αsyn group. Similarly, GSDME (*P* = 0.0002, Fig. 2C), IL-1β (*P* = 0.0009, Fig. 2C), IL-18 (*P* = 0.0336, Fig. 2C) and IL-6 (*P* = 0.0075, Fig. 2C) were found elevated in SH αsyn group. However, increased levels of miR-675-5p before NM-Sal treatment has reduced IL-6 (*P* = 0.0036, Fig. 2D), NLRP3 (*P* = 0.0004, Fig. 2D) level in SH-gfp group, and downregulated GSDME (*P* = 0.0095, Fig. 2D), IL-18 (*P* = 0.0354, Fig. 2D) in PD neuron models at different levels. Downregulated miR-1247-5p before NM-Sal treatment increased HMGB1 (*P* = 0.0023, Fig. 2E) in PD neuron models and NLRP3 (*P* = 0.0030, Fig. 2E) in SH gfp group.

Our previous studies have verified that miRNAs significantly influence the degenerative process of PD by their normal cross-talk with circRNA and lncRNA [22, 23]. Through literature surveys and databases, we identified circSLC8A1 and lncH19 have interaction with the miR-675-5p and miR-1247-5p. Hence, we analyzed the expression levels of these ncRNAs in the serum of PD patients to explore whether they can act as diagnostic biomarkers.

### Cohort demographics

Table 1 summarizes the demographic and clinical data of all participants. There were no significant differences in age and gender between PD and the control cohort.

**Table 1:** The demographic distribution between study cohorts

Demographic	ncRNA		
	Ctl, *n* = 106	PD, *n* = 104	*P* value
Age,mean(SEM)	64.04 (1.198)	66.2 (1.182)	0.2000
Sex,M/F	48/58	58/46	0.1298
Disease duration (years)	NA	7.363 ± 0.8551	NA
LEDD total (mg)	NA	510.5 ± 26.86	NA
H&Y stages	NA	2.466 ± 0.08486	NA

LEDD, levodopa equivalent daily dose; H&Y stages, Hoehn-Yahr stages; NA, not available; PD, Parkinson disease; Ctl, control; ncRNA, noncoding RNA

### Serum non-coding RNAs expression

**Fig. 3:**
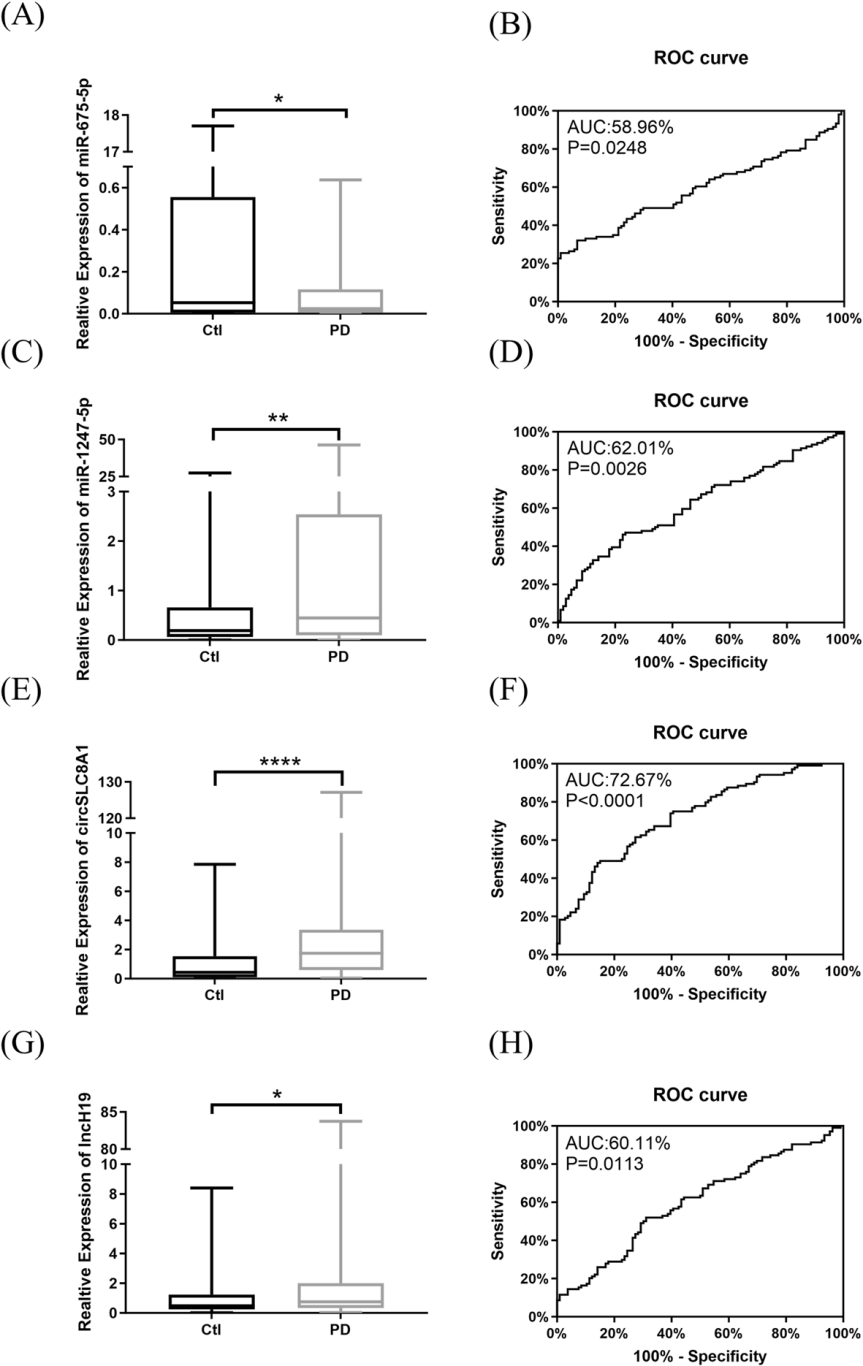
Non-coding RNA levels in all participants. The expression levels of miR-675-5p (**A**), miR-1247-5p (**C**), circSLC8A1 (**E**), and lncH19 (**G**) were measured by RT-qPCR. The delta-delta cycle threshold value (2^-ΔΔCt^) method was used to analyze the results relative to the internal parameters, and the results were compared with the age-matched healthy control cohort (PD, *n* = 104; Ctl, *n* = 106). The midline in the box and whisker plots depicts the median, with upper and lower limits representing maximum and minimum values. ROC curve analysis of miR-675-5p (**B**), miR-1247-5p (**D**), circSLC8A1 (**F**), and lncH19 (**H**) relative to healthy controls (PD, Parkinson disease; Ctl, control; miR, microRNA; circ, circular RNA; lnc, long-noncoding RNA; ROC, receiver operating characteristic; *P < 0.05;**P < 0.01; ****P < 0.0001)

Our RT-qPCR results revealed significantly different expression levels of miR-675-5p, miR-1247-5p, circSLC8A1 and lncH19 in the PD patients as compared with controls. Relative to controls, miR-675-5p concentrations for the PD patients was 0.09042 (*P* = 0.0249, Fig. 3A), whereas miR-1247-5p, circSLC8A1 and lncH19 levels for the PD patients was 2.773 (*P* = 0.0026, Fig. 3C), 4.485 (*P* < 0.0001, Fig. 3E), 3.715 (*P* = 0.0114, Fig. 3G).

### Receiver operating characteristic analyses

Receiver-operating characteristic (ROC) analysis suggested that miR-675-5p levels distinguish PD patients from controls with an area below the curve of 58.96% (95% confidence interval [CI] 51.15–66.78). When the cutoff value was 18.38, it showed 93.27% Sensitivity and 32.71% Specificity (Fig. 3B); the serum miR-1247-5p cutoff value of 0.09451 had a modest 47.12% Sensitivity and 76.42% Specificity for distinguishing between patients with PD and controls, with an area under the curve of 62.01% (95% confidence interval [CI] 54.43—69.59; Fig. 3D).

For circSLC8A1 and lncH19, the area under curves was 72.67% (95% confidence interval [CI] 65.93–79.41) and 60.11% (95% confidence interval [CI] 52.47–67.76), respectively. Compared to the control cohort, the cutoff value of circSLC8A1 was 0.31, its sensitivity was 75%, and specificity was 59.43% (Fig. 3F). At the cutoff value of was 0.1376, lncH19 sensitivity was 51.92%, and specificity was 68.87% (Fig. 3H).

### Effects of medications

In the subgroup of PD patients, the mean LEDD was 510.5 (SEM = 26.86) mg. miR-675-5p (*P* = 0.5315), miR-1247-5p (*P* = 0.8259), circSLC8A1 (*P* = 0.0538), lncH19 (*P* = 0.2391) expression levels were not correlated to LEDD. A detail can be obtained in the Additional file 2: Table S2.

### Progression of PD

**Fig. 4:**
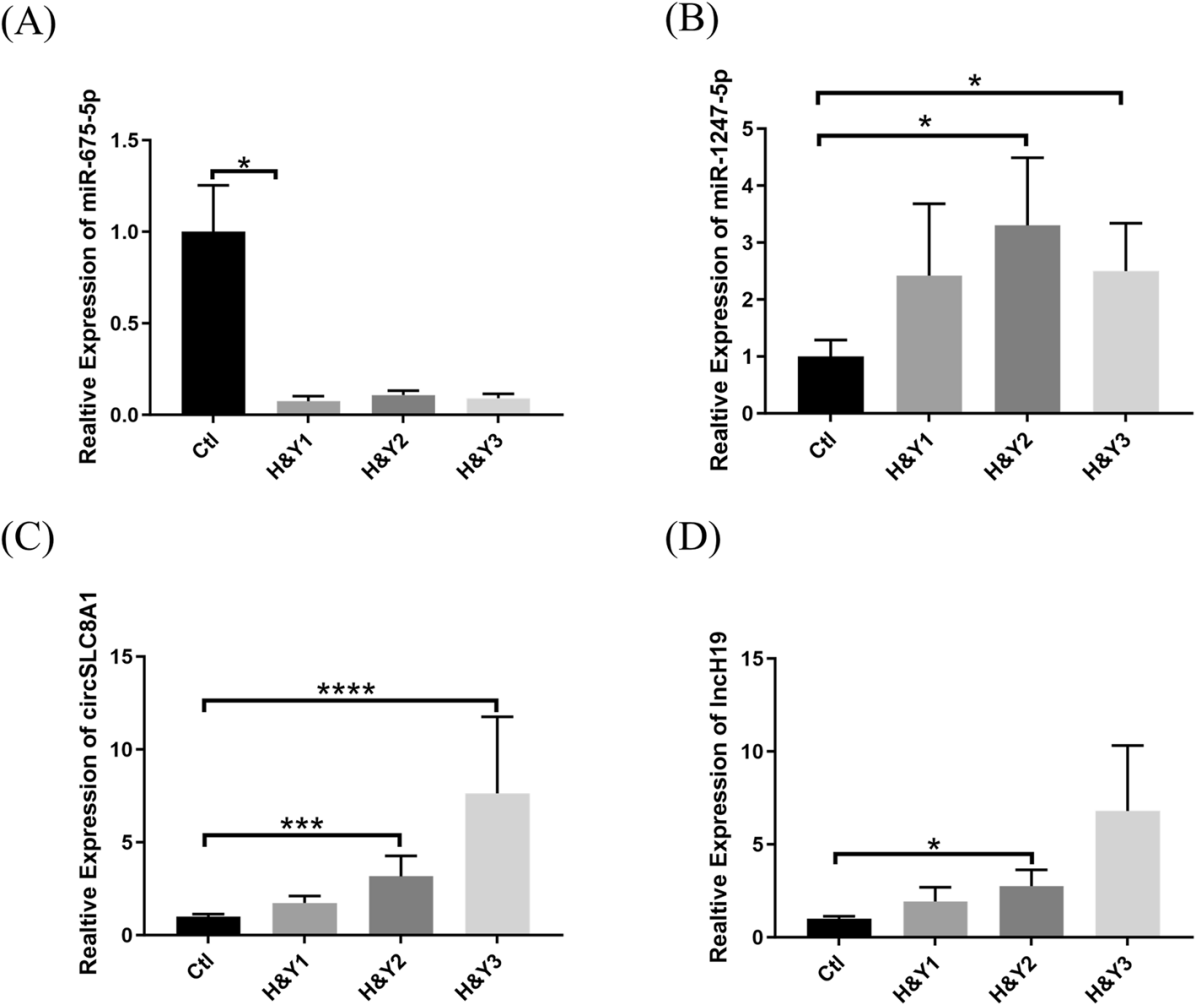
The role of non-coding RNAs in PD H&Y stage and disease duration. The expression levels of miR-675-5p (**A**), miR-1247-5p (**B**), circSLC8A1 (**C**) and lncH19 (**D**) in each H&Y stage were measured by RT-qPCR. The delta-delta cycle threshold value (2^-ΔΔCt^) method was used to analyze the results relative to endogenous and experimental controls (H&Y1, *n* = 20; H&Y2, *n* = 46; H&Y3, *n* = 31; Ctl, *n* = 106). (Ctl, control; H&Y, Hoehn and Yahr; miR, microRNA; circ, circular RNA; lnc, long noncoding RNA; *P < 0.05; ***P < 0.001; ****P < 0.0001)

Clinically, PD patients were divided into different H&Y stages according to the severity of their pathological condition. Increased H&Y stage remained proportional to the decreased cognitive levels in PD patients, which is presumably due to the malfunctioning of the dopaminergic neurons [52]. For early diagnosis, patient samples were collected at H&Y1,2,3 stages (*n* = 97); however, the age and gender of PD patients in these H&Y stages were not significantly different from that of the control cohort. For instance, patients in H&Y stage 1 showed significantly downregulated expression of miR-675-5p levels (*P* = 0.0420, Fig. 4A); whereas PD patients in H&Y stages 2 and 3 showed significantly higher serum miR-1247-5p concentrations relative to controls (*P* = 0.0385 and *P* = 0.0271 respectively; Fig. 4B). No remarkable difference was observed in other stages. However, in comparison with non-PD cohorts, serum circSLC8A1 levels of PD patients at H&Y stage 2, 3 displayed significant differences in disease progression (*P* = 0.0002 and *P* < 0.0001 respectively, Fig. 4C); whereas H&Y stage 2 PD patients showed a significant difference in the lncH19 levels as compared to control cohort (*P* = 0.0211, Fig. 4D).

The estimated disease duration was not remarkably correlated with expression levels of miR-675-5p (*P* = 0.8142), miR-1247-5p (*P* = 0.7154), circSLC8A1 (*P* = 0.4986) or lncH19 (*P* = 0.7123). An additional Table file shows this in more detail (see Additional file 3).

### Downstream proteins

**Fig. 5:**
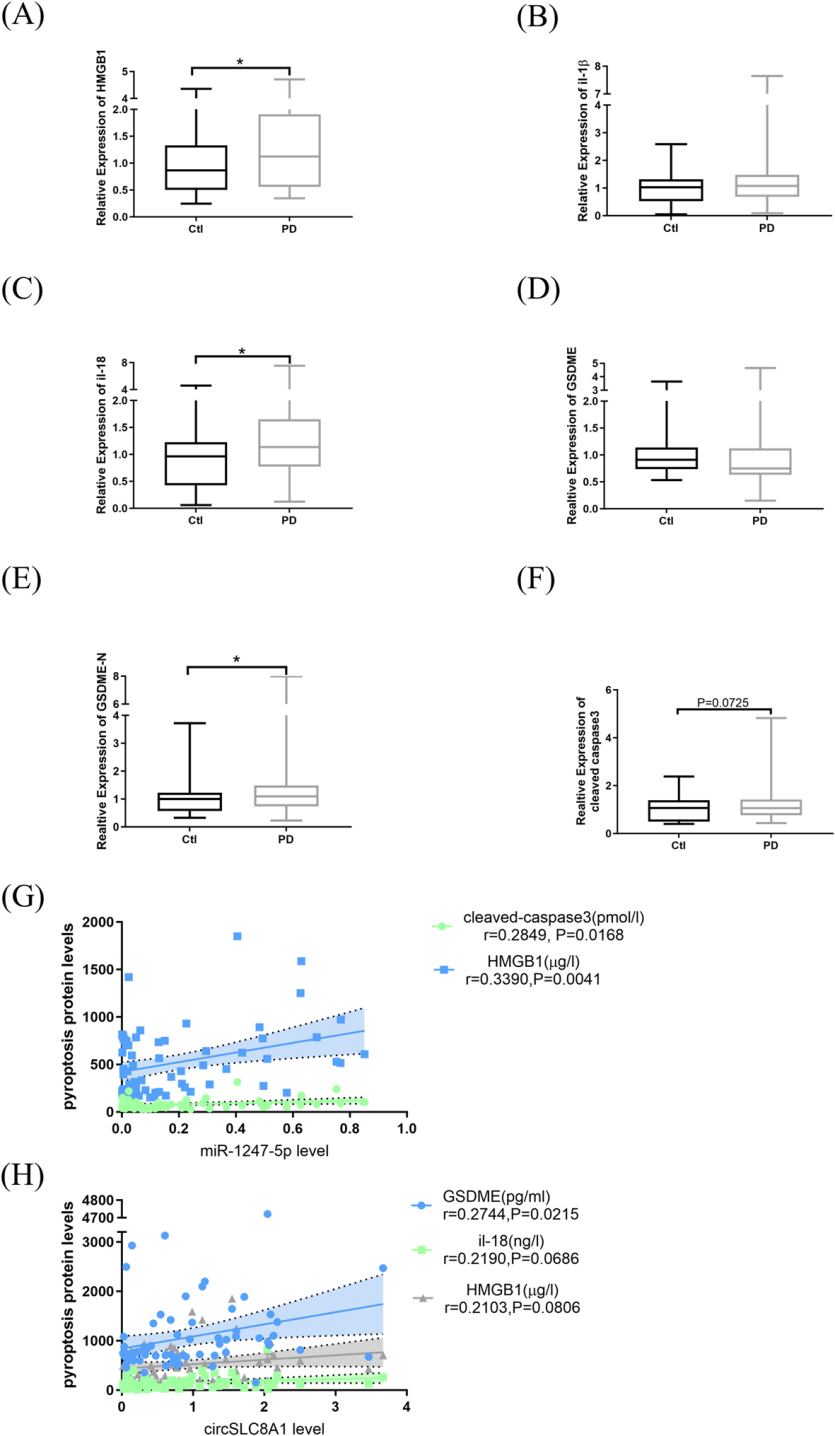
Pyroptosis proteins and non-coding RNAs in the subset of PD patients. Mean expression levels of HMGB1 (**A**), IL-1β (**B**), IL-18 (**C**), GSDME (**D**), GSDME-N (**E**) and cleaved caspase3(**F**) were determined by ELISA and relative to the control cohort (PD, *n* = 70; Ctl, *n* = 54). The midline in the box and whisker plots depicts the median, with upper and lower limits representing maximum and minimum values. Correlation analysis between miR-1247-5p with cleaved caspase3, HMGB1 (**G**); circSLC8A1 with GSDME, IL-18, and HMGB1 expression levels (**H**) and shaded areas represent 95% confidence intervals (Ctl, control; PD, Parkinson patients; miR, microRNA; circ, circular RNA; lnc, long non-coding RNA; *P < 0.05)

In a randomly selected subset of the study population (70 patients and 54 controls), serum pyroptosis-associated protein HMGB1 (*P* = 0.0429, Fig. 5A) and IL-18 (*P* = 0.0199, Fig. 5C) were significantly increased in PD patient’s serum compared to control. The results remained consistent with the results of B Aslıhan and A Aziza [53, 54]. Nonetheless, the mean levels of IL-1β (*P* = 0.1964, Fig. 5B) and GSDME (*P* = 0.1496, Fig. 5D) showed no significant difference relative to the healthy control cohort. Notably, the average level of GSDME-N (*P* = 0.0413, Fig. 5E) and cleaved caspase3 (*P* = 0.0725, Fig. 5F) was robustly increased, suggesting the Caspase3-GSDME pathway may be switched on and lead to dopaminergic neurons pyroptosis with inflammatory factors released into the extracellular environment.

On the other hand, positive correlations with pyroptosis marker cleaved caspase-3 protein and HMGB1 concentrations were found against miR-1247-5p level (*P* = 0.0168 and *P* = 0.0041 respectively, Fig. 5G). circSLC8A1 was positively correlated with the pyroptosis-related protein GSDME (*P* = 0.0215, Fig. 5H). Meanwhile, inflammatory factor IL-18 and HMGB1 showed near positive correlations with circSLC8A1 (*P* = 0.0686 and *P* = 0.0806, respectively, Fig. 5H). However, no correlation between other ncRNAs and pyroptosis-related proteins/inflammatory factors was found in our research (data not shown). Additionally, the samples used in the randomly selected subset were representative of the overall population, having no significant differences in age and sex between the PD and non-PD cohorts.

### Multi-biomarker combination

**Fig. 6:**
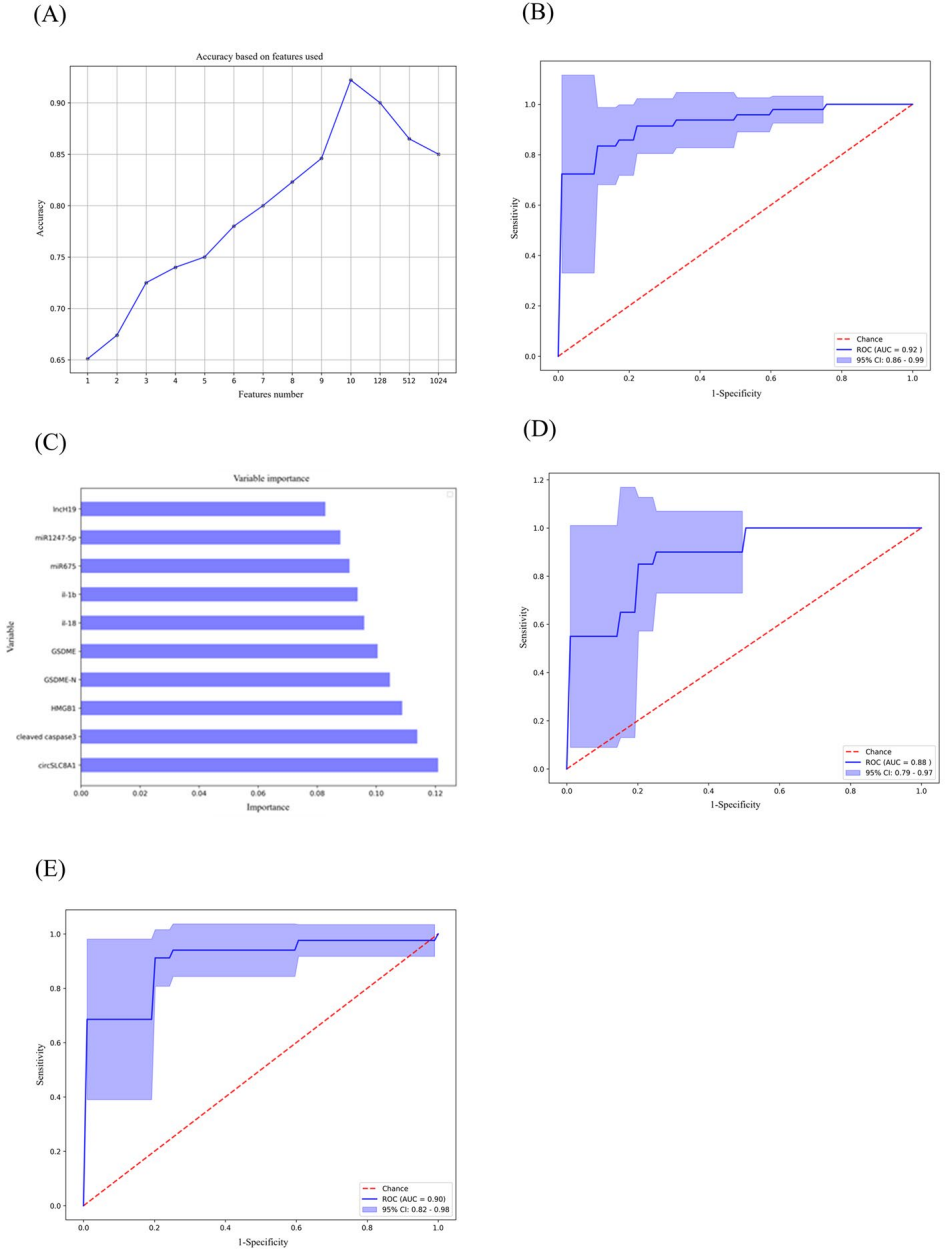
Combined biomarkers constructed with the machine learning algorithm. Ranking the contribution of 10 biomarkers to the construction of predictive models (**A**). ROC analysis prediction model with random forest in test subset (**B**). The importance of 10 variables to prediction model (**C**). ROC analysis prediction model with random forest in H&Y stage ≤ 2 subsets (**D**) and disease course ≤ 5 years subset (**E**). The shaded region of the ROC curve represents the 95% confidence interval (miR, microRNA; circ, circular RNA; lnc, long non-coding RNA; ROC, receiver operating characteristic; AUC, area under the curve)

According to evidence from dysregulated miRNAs and lncRNAs, machine learning models could serve as a great alternative to assist in clinical diagnosis and make it simpler to identify neurological disorders in their early stages [55]. In our study, we found several serum biomarkers, including miR-675-5p, miR-1247-5p, circSLC8A1, lncH19, and pyroptosis-related proteins of PD and trained different classifiers with logistic regression and random forest approaches based on the training subset (56 PD and 43 healthy cases) in the manner of single or multiple biomarkers. Subsequently, the capacity to predict models of the random forest was higher than that of logistic regression (Data not shown). Of all obtained classifiers from the random forest, the resulting predictive classifier with the optimal accuracy (AUC: 92%, 95% confidence interval [CI] 86–99) has covered ten biomarkers (14 PD and 11 healthy cases, Fig. 6A, B). We ranked the ten biomarkers depending on their contribution to diagnosing PD patients, and lncH19 had the lowest effort (Fig. 6C).

Based on the above results, we further explored whether the panel of 10 biomarkers with a random forest algorithm could still ensure high diagnostic accuracy for early-stage PD patients (H&Y stage ≤ 2 or disease course ≤ 5 years). The accuracy rate of our obtained model reached 88%(95% confidence interval [CI] 79–97) in the H&Y stage ≤ 2 subsets and 90% (95% confidence interval [CI] 82–98) in the disease course ≤ 5 years subset separately (Fig. 6D, E).

## Discussion

Parkinson’s disease presents a diagnostic challenge due to its complex etiology and the lack of definitive biomarkers for early detection. The pathogenesis of PD involves intricate molecular mechanisms, including abnormal protein aggregation, neuroinflammation and neuronal cell death [6, 7, 56]. Convincing evidence has shown that understanding the early molecular events in PD has become crucial for timely intervention and better disease management [10]. Among these, pyroptosis, a form of programmed cell death, has gained attention as an early molecular event triggering PD pathogenesis [37, 57, 58]. Pyroptosis is characterized by proinflammatory cell death, often triggered by cellular insults. In PD, pyroptosis is believed to be linked to the release of inflammatory mediators and the activation of glial cells, contributing to the neuroinflammatory state seen in the disease [59]. It is proposed that detecting pyroptosis at an early stage is essential for comprehending disease progression and developing targeted therapies [59–61]. Numerous studies have shown that the various pyroptosis proteins, including IL-1β, IL-2, IL-6, IL-10, TNF-α, NLRP3 and SOD1, among others, can be utilized as peripheral inflammatory biomarkers in PD patients. However, these findings are constrained by issues with specificity, sensitivity, variability and a lack of causal relationships, making them neither accurate nor ideal for PD diagnosis (Detail is mentioned in Additional file 5) [54, 57, 62, 63]. Recently, various miRNAs have been identified as early detectable peripheral biomarkers in PD and are under clinical trials owing to their small size, stability and endogenous nature [25, 64]. Accumulating evidence has shown that several ncRNAs regulate the initiation of pyroptosis by NLRP3 in PD invitro MPP + models (Detail is mentioned in Additional file 5) [58, 62, 65–68]. However, scare studies have reported ncRNAs in the serum or plasma samples of the PD patients, limiting the effectiveness of the candidate ncRNAs as potential biomarkers. Moreover, the underlying mechanism involving the interplay of ncRNAs in pyroptosis-mediated pathways in PD is still a mystery. Thus, it is proposed that identifying specific ncRNAs pattern in pyroptosis-mediated pathways from the body fluids of the PD patients have more potential to serve as biomarkers for detecting early PD pathology.

Therefore, the novelty of the present study delves into the emerging diagnostic potential of the pyroptosis-mediated ncRNAs biomarker pattern for PD. Through a comprehensive investigation encompassing in-vitro and in-vivo PD models, as well as clinical samples from PD patients, we identified and analyzed specific miRNAs (miR-675-5p and miR-1247-5p) and their regulatory role in pyroptosis and neuroinflammation. Furthermore, we explored the diagnostic efficacy of these miRNAs, in conjunction with circSLC8A1 and lncH19, in discriminating PD patients from healthy controls. To the best of our knowledge, this is the first study to highlight the importance of miR-675-5p and miR-1247-5p against pyroptosis in PD, signifying their potential as biomarkers for early PD detection and intervention.

Our initial investigation involved the examination of miR-675-5p and miR-1247-5p in PD cell line and rat models. Our results showed dysregulated expression of miR-675-5p and miR-1247-5p in response to neurotoxin NM-Sal and α-syn, implicating their involvement in neurological functioning and promoting PD pathogenesis. Notably, miR-675-5p displayed contrasting expression patterns under the influence of NM-Sal alone versus NM-Sal combined with α-syn, suggesting a potential synergistic effect of these pathogenic variables. Conversely, miR-1247-5p levels were consistently elevated in the presence of NM-Sal and α-syn. Previously, miR-675-5p and miR-1247-5p have been reported to play a significant role in triggering abnormal cellular functions in various neurological malignancies [28, 29, 31, 33]. However, the pathological mechanisms involved in these neurological malignancies are still under investigation, hinting that some molecular mysteries are involved in triggering the pathogenic mechanisms in neurological disorders, especially PD. This hypothesis was further supported by recent studies demonstrating that dysregulated expression of miR-675-5p is involved in neuronal damage by stimulating neuronal apoptosis and promoting glioma cell proliferation [32]. Similarly, early brain injury, which is a common medical manifestation of the majority of central nervous issues, has revealed dysregulated expression of miR-675-5p that resulted in neuronal damage through triggering H19-miR-675-P53 apoptosis [29, 69]. Additionally, dysregulated expression of miR-1247-5p in the cortex region of Huntington’s disease patients was reported to implicate essential neurological functions, such as neuronal differentiation, neurite outgrowth, cell death and survival [30, 70]. However, in human astroglioma cells, lowered levels of miR-1247-5p promoted glioma cells proliferation and migration, whereas higher levels of miR-1247-5p functioned as a tumour suppressor and exhibited neuroprotective characteristics [33, 34, 71]. Hence, based on the previous studies supporting the characteristic feature of miR-675-5p and miR-1247-5p involved in regulating vital neurological functions, it is proposed that the complex interplay of the miR-675-5p and miR-1247-5p might be involved in the pathogenesis of PD: however, the underlying mechanisms regulated by miR-675-5p and miR-1247-5p in PD pathogenesis is still unknown.

To further probe the underlying mechanism regulated by miR-675-5p and miR-1247-5p in PD pathogenesis, we performed bioinformatics analysis and identified potential interactions of miR-675-5p with GSDME and miR-1247-5p with HMGB1, suggesting their involvement in pyroptosis-related pathways. Subsequent experimental validation confirmed the regulatory effects of miR-675-5p and miR-1247-5p on the expression of GSDME and HMGB1, respectively. GSDME, as one of the members of the Gasdermin family, is initially reported in cancer cell lines where it mediates pyroptosis after being cleaved by caspase-3 [40, 43]. GSDME acts like a switch molecule for the transformation between apoptosis and pyroptosis, such that overexpressed GSDME triggers tumour cell death through caspase-3-dependent pyroptosis, whereas its low levels transit cell death mode to apoptosis [42]. For instance, in breast cancer, GSDME has activated DOX-induced pyroptosis in the caspase-3-dependent reactions through the ROS/JNK signaling pathway [72, 73]. Similarly, Dong et al. reported that the microglial inflammasome/caspase-1/GSDMD pathway and the neuronal caspase-3/GSDME signalling pathway are major molecular events underlying pyroptosis [74]. However, the functional significance of the neuronal capsase-3/GSDME signalling pathway in the CNS remains a mystery. Recent studies have shown that higher levels of GSDME play a significant role in the progression of neurodegenerative diseases by translocating into neuronal mitochondria to cause mitochondrial depolarization and neuronal damage [75, 76]. Studies on ischemia–reperfusion have shown that elevated lncH19 expression would significantly boost NLRP3/6 inflammasome imbalance, causing pyroptosis in the microglia and neurons [77]. Meanwhile, extensive literature has confirmed that miR675 helps lncH19 to perform and execute physiological functions [78, 79]. Nevertheless, GSDME's potent role associated with proptosis in PD is still unclear; therefore, it is hypothesized that dysregulated expression of miR-675-5p might disturb GSDME levels and promote PD pathogenesis. Additionally, damaged Caspase3/GSDME cells also generate HMGB1, which functions as a DAMP to exacerbate the inflammatory response [80]. Accumulating evidence has shown that HMGB1, as a key promoter of neuroinflammation, is believed to be involved in the neurodegenerative process of various neurological disorders, including PD [81]. Interestingly, our results also confirmed previous studies, implying that dysregulated levels of HMGB1 as potent target of miR-1247-5p have exerted modulatory effects on neuroinflammation markers, NLRP3, IL-1β, IL-18, and IL-6, reinforcing their potential roles in mitigating pyroptosis and neuroinflammation in PD.

Additionally, to evaluate the potential of miR-675-5p and miR-1247-5p as diagnostic biomarkers in PD patients, we evaluated the expression pattern of miR-675-5p, miR-1247-5p and their linking ncRNAs such as circSLC8A1, and lncH19 in the serum of PD patients. Our findings demonstrated the dysregulated expression of miR-675-5p, miR-1247-5p, circSLC8A1, and lncH19 in the serum of PD patients compared to healthy controls. The receiver operating characteristic (ROC) analysis further validated the diagnostic potential of miR-675-5p, miR-1247-5p with circSLC8A1 and lncH19 exhibiting notably high sensitivity and specificity. In line with our findings, a previous study by Cheng et al. reporting the regulatory role of CircSV2b in controlling the oxidative stress via miR-5107-5p-Foxk1-Akt1 axis in PD supported the combinational use of ncRNA for the diagnosis of PD [82]. Similarly, a recent study by Lin et al. revealed the promising role of lncRNA MEG8 in ameliorating the neuroinflammation in PD through the miR-485-3p/FBXO45 axis [83]. In another study, circSLC8A1 levels in the substantia nigra of PD patients were found in higher levels [84], and coincidentally, our bioinformatics-prediction (http://www.circbank.cn/) revealed that circSLC8A1 is closely linked with miR-1247-5p. Hence, based on previous reports, the findings obtained in our study underscore the promise of these ncRNAs in improving PD diagnosis, especially when used in combination. Moreover, to analyze the multi-biomarker approach for enhanced diagnostic accuracy, we employed machine learning algorithms and devised a predictive classifier combining miR-675-5p, miR-1247-5p, circSLC8A1 and lncH19 with pyroptosis-associated proteins. The resulting model exhibited high accuracy in distinguishing PD patients from controls, even in early-stage PD (H&Y stage ≤ 2) and within a limited disease duration (≤ 5 years), highlighting the potential of combining ncRNAs and pyroptosis-associated proteins for a robust diagnostic tool, aligning with previous research emphasizing the power of multi-biomarker approaches in disease diagnosis [85].

**Fig. 7:**
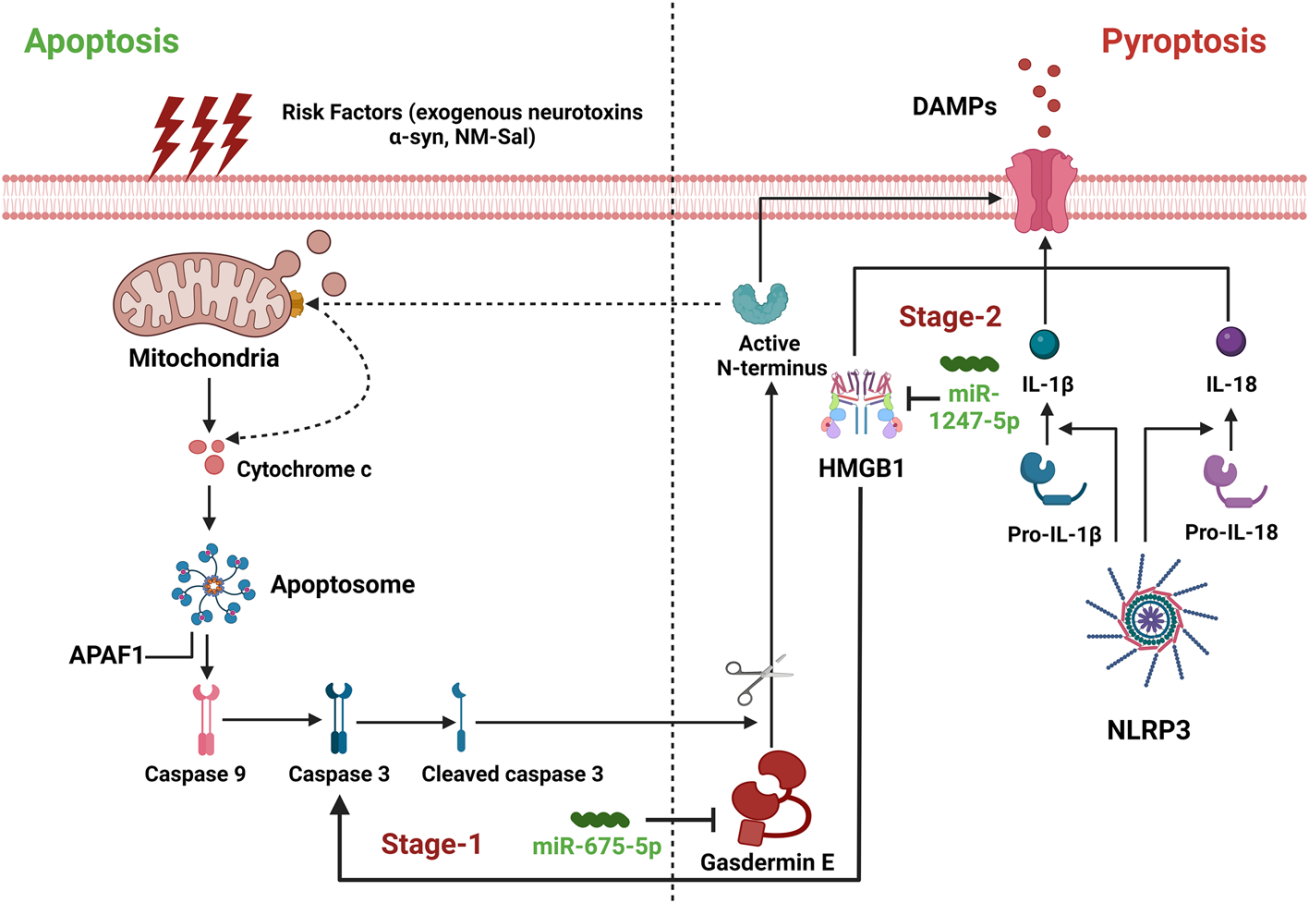
The potential role of miR-675-5p and miR-1247-5p in the pathogenesis of PD

**Fig. 8:**
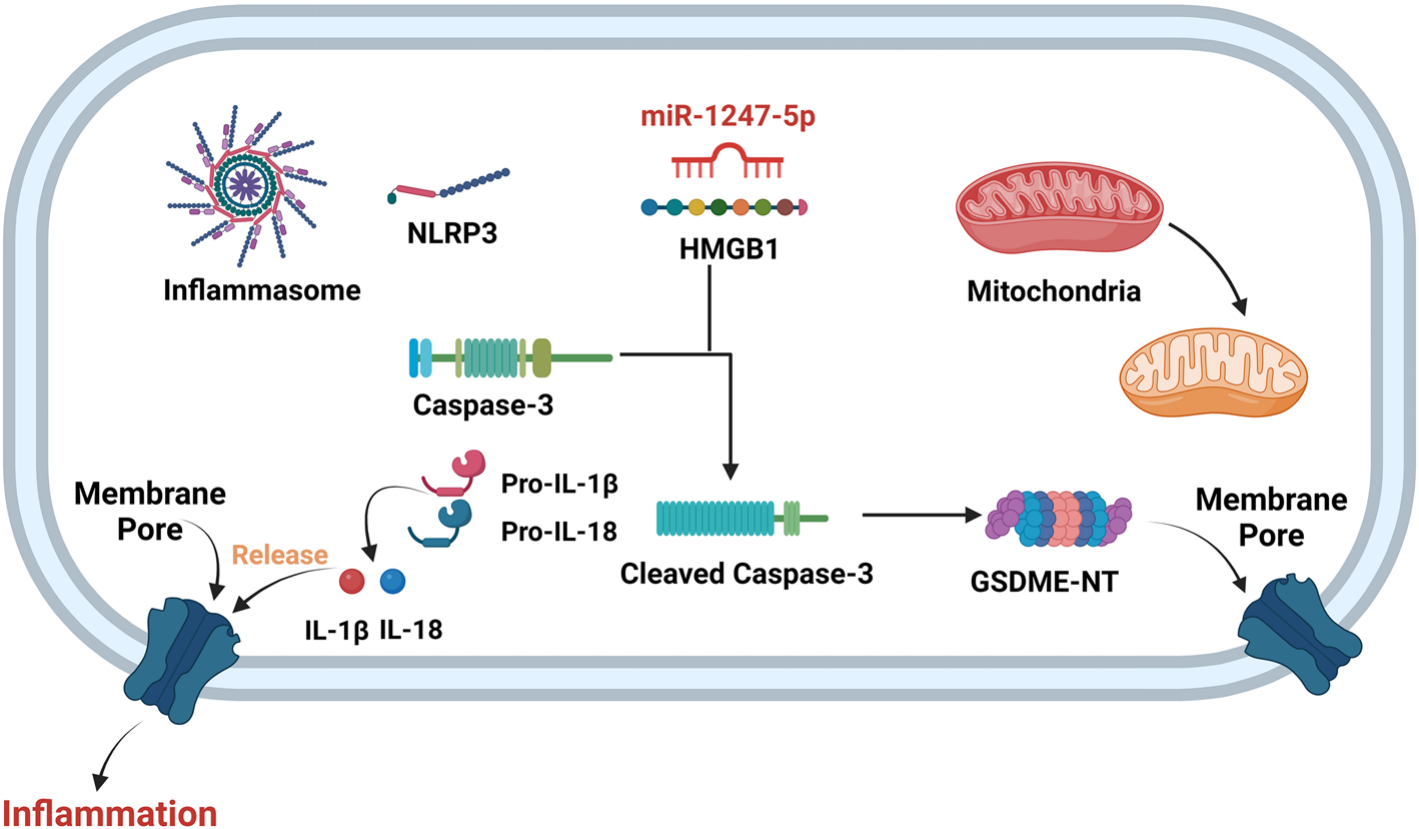
The potential role of miR-1247-5p in the pathogenesis of PD

Overall, the findings from the serum of PD patients suggest that the miR-675-5p and miR-1247-5p may be in charge of the GSDNE’s conversion from appoptosis to pyroptosis. In order to illustrate how dysregulated miR-675-5p and miR-1247-5p interact to cause PD, we presented the model depicted in Fig. 7, showing the interplay of miR-675-5p and miR-1247-5p regulating GSDME's transition from apoptosis to pyroptosis. In the early stages of PD (H&Y stage 1), exposure to external risk factors, including α-syn, NM-Sal, and others, induces GSDME-N to release Cyt C from the mitochondria. This leads caspase-3 to get activated and cleave GSDME, which causes neuronal apoptosis and causes neuronal damage. It is well known that GSDME works both upstream and downstream of caspase-3 to encourage caspase-3 cleavage and cause pyroptosis. Thus, under conditions of neuronal toxicity, neurons produced an adaptive response by decreasing miR-675-5p, as seen in our PD models, which activated GSDME. Higher levels of GSDME cause neuronal cells to undergo the transition from apoptosis to pyroptosis and trigger brain immune responses against risk factors. Additionally, HMGB1 is produced by damaged Caspase3/GSDME cells and acts as a DAMP to amplify the inflammatory response. Therefore, it illustrates a compelling explanation for how neuroinflammation and pyroptosis worsen in PD. However, in middle-stage PD (H&Y stage 2, 3), neuroinflammation caused by an over-activated immune response inhibit neuronal survival. As a result, neurons upregulate miR-1247-5p levels as a defensive response to lower HMGB1 and NLRP3 levels, which ultimately lowers neuroinflammation. It is believed that miR-1247-5p targets HMGB1 to inhibit the Caspase3-GSDME signaling pathway and slow neuronal pyroptosis in PD, as shown in Fig. 8.

However, the current study does have certain limitations. (i) According to our data, miR-1247-5p targets HMGB1, but their levels in the serum of PD patients are positively correlated, hence suggesting more investigations to identify miR-1247-5p’s potential role in the etiology of Parkinson's disease; (ii) Additionally, all participants are from the same region, and there is no replication queue, proposing that further sampling from different ethnic groups may give a better understanding of the potential role of miR-675-5p and miR-1247-5p as diagnostic biomarkers; (iii) The relative quantification approach of qPCR restricts comparisons of the absolute quantities of ncRNA between our work and other studies, which can theoretically be solved if other quantification approaches, like RNA-Seq or absolute quantification using standards, can be utilized; (iv) Compared to ncRNAs, the sample size for pyroptotic proteins was relatively small, which can be resolved by increasing the sample size, that may presumably enhance the statistical power of the analysis but also provide more robust results for pyroptotic proteins; and (v) Although random forest algorithms may accurately classify and manage missing data, still they have the disadvantage of being complicated and challenging to comprehend.

In conclusion, this study unveiled the occurrence of pyroptosis in patients with PD, which is indicated by the serum levels of miR-675-5p, miR-1247-5p, circSLC8A1, and lncH19. This not only insinuates their potential role in the pathogenesis and diagnostic stratification of PD, but also suggests their potential as accessible, minimally invasive biomarkers for diagnosing and evaluating PD. Incorporating these ncRNAs into a comprehensive multi-biomarker approach could significantly enhance early PD diagnosis and therapies, marking a considerable stride forward in the realm of PD. Absolutely, in the future, the synergistic utilization of advanced AI techniques could potentially unravel the complex relationships between these ncRNAs and other factors contributing to PD pathogenesis [86, 87]. This integrated strategy might lead to the identification of novel therapeutic targets or intervention strategies, thus creating a pathway for more effective and individualized treatment options for Parkinson’s disease.

### Supplementary Information

**Additional file 1: Table S1.** Primer sequences for ncRNAs and mRNAs.

**Additional file 2: Table S2.** Correlation coefficients between LEDD (mg) and ncRNAs.

**Additional file 3: Table S3.** Correlation coefficients between disease duration (year) and ncRNAs.

**Additional file 4: Figure S1.** NM-Sal dose for PD neuron model construction.

**Additional file 5:** **Table S4.** Studies of pyroptosis related ncRNAs as potential biomarkers for PD. **Table S5.** Studies of pyroptosis related proteins as potential biomarkers for PD.

**Additional file 6: Figure S2.** Transfection validation of miRNAs.

## Data Availability

Data not provided in the article because of space limitations may be shared (anonymized) at the request of any qualified investigator for purposes of replicating procedures and results.

## Abbreviations

PD: Parkinson disease
ncRNA: Non-coding RNA
RT-qPCR: Reverse transcriptase quantitative polymerase chain reaction
SNpc: Substantia nigra pars compacta
CSF: Cerebrospinal fluid
CNS: Central nervous system
miRNA: MicroRNA
circRNA: Circular RNA
lncRNA: Long non-coding RNA
DAN: Dopaminergic neuron
GSDMD: Gasdermin D
GSDME: Gasdermin E
GSDME-N: GSDME-N terminal
NMDAR: N-methyl-D-aspartate receptor
NM-Sal: 1(R)-2(N)-dimethyl-6,7-dihydroxy-1,2,3,4-tetrahydroisoquinoline
LEDD: Levodopa equivalent daily dose
Ctl: Control
ROC: Receiver operating characteristic
AUC: Area under the curve
