# Supplementary material for: The pyroptosis mediated biomarker pattern: an emerging diagnostic approach for Parkinson’s disease
Source: Cell Mol Biol Lett. 2024 Jan 3;29:7. doi: 10.1186/s11658-023-00516-y (PMC10765853; doi:10.1186/s11658-023-00516-y)
Supplement: Supplementary file 1 — Additional file 1: Table S1. Primer sequences for ncRNAs and mRNAs. [file 11658_2023_516_MOESM1_ESM.docx]

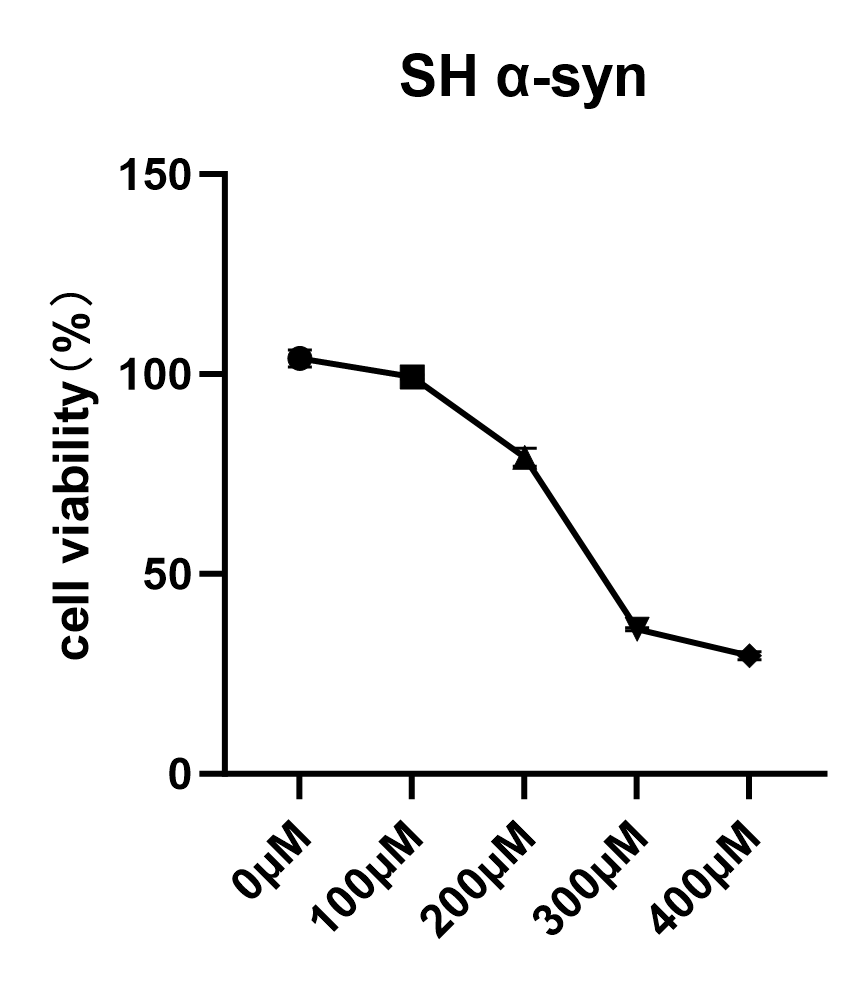

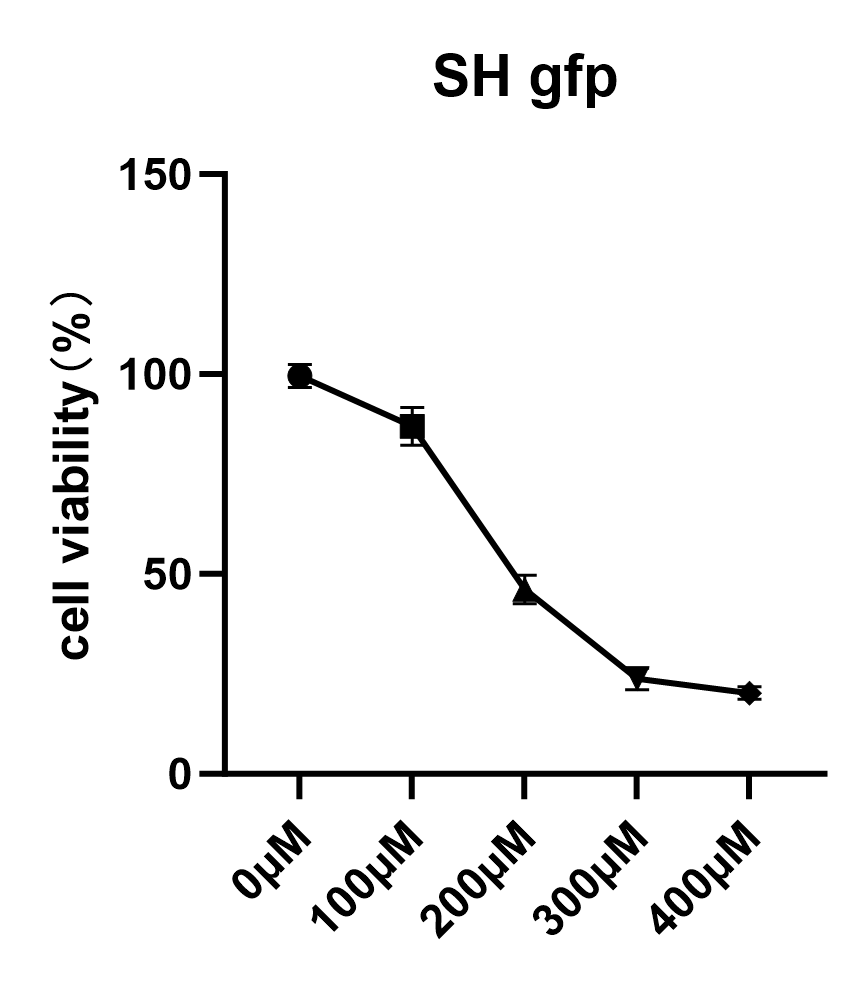
(A) (B)

Additional Fig 1 NM-Sal dose for PD neuron model construction

Results of CCK-8 assays indicated that 200μM NM-Sal can simulate chronic injury of PD in SH gfp and SH αsyn cells well.
