# Supplementary material for: The pyroptosis mediated biomarker pattern: an emerging diagnostic approach for Parkinson’s disease
Source: Cell Mol Biol Lett. 2024 Jan 3;29:7. doi: 10.1186/s11658-023-00516-y (PMC10765853; doi:10.1186/s11658-023-00516-y)
Supplement: Supplementary file 2 — Additional file 2: Table S2. Correlation coefficients between LEDD (mg) and ncRNAs. [file 11658_2023_516_MOESM2_ESM.docx]

Additional table 2. Correlation coefficients between LEDD(mg) and ncRNAs

| ncRNA | r | P value |
| --- | --- | --- |
| miR-675-5p | 0.0621 | 0.5315 |
| miR-1247-5p | 0.0218 | 0.8259 |
| circSLC8A1 | 0.1897 | 0.0538 |
| lncH19 | -0.1165 | 0.2391 |

(LEDD, levodopa equivalent daily dose ; miR, microRNA; circ,circular RNA;lnc,long-noncoding RNA；ncRNA, non-coding RNA)
