# Supplementary material for: The pyroptosis mediated biomarker pattern: an emerging diagnostic approach for Parkinson’s disease
Source: Cell Mol Biol Lett. 2024 Jan 3;29:7. doi: 10.1186/s11658-023-00516-y (PMC10765853; doi:10.1186/s11658-023-00516-y)
Supplement: Supplementary file 3 — Additional file 3: Table S3. Correlation coefficients between disease duration (year) and ncRNAs. [file 11658_2023_516_MOESM3_ESM.docx]

Additional table 3. Correlation coefficients between disease duration(year) and ncRNAs

| ncRNA | r | P value |
| --- | --- | --- |
| miR-675-5p | -0.0233 | 0.8142 |
| miR-1247-5p | -0.0362 | 0.7154 |
| circSLC8A1 | 0.0671 | 0.4986 |
| lncH19 | 0.0366 | 0.7123 |

(miR, microRNA; circ,circular RNA;lnc,long-noncoding RNA; ncRNA,noncoding RNA)
