# Supplementary material for: The pyroptosis mediated biomarker pattern: an emerging diagnostic approach for Parkinson’s disease
Source: Cell Mol Biol Lett. 2024 Jan 3;29:7. doi: 10.1186/s11658-023-00516-y (PMC10765853; doi:10.1186/s11658-023-00516-y)
Supplement: Supplementary file 5 — Additional file 5: Table S4. Studies of pyroptosis related ncRNAs as potential biomarkers for PD. Table S5. Studies of pyroptosis related proteins as potential biomarkers for PD. [file 11658_2023_516_MOESM5_ESM.docx]

Additional table 4. Studies of pyroptosis related ncRNAs as potential biomarkers for PD

| sample | model | Biomarkers | Reference |
| --- | --- | --- | --- |
| NA | neuron model treated with MPP+ | miR-135b: candidate biomarkers for PD | [1-3] |
| NA | neuron model treated with MPP+ | miR-326: candidate biomarker for PD | [4, 5] |
| serum/plasma/ cerebral fluid | neuron model treated with conditioned medium | miR-124-3p: candidate biomarker for PD | [6, 7] |

Additional table 5. Studies of pyroptosis related proteins as potential biomarkers for PD

| sample | Participants | Biomarkers | Reference |
| --- | --- | --- | --- |
| serum | early PD (58), Ctl (20) | IL-1β, IL-2, IL-6, IL-10, tumor necrosis factor-  α, and high-sensitivity C-reactive protein: candidate biomarkers for early PD | [8] |
| serum | PD (30), Ctl(30) | HMGB1, hs-CRP: candidate biomarkers for PD | [9] |
| serum | baseline cohort: PD (27), Ctl (16)  Year 1 Cohort: PD (20), Ctl (15) | NLRP3,IL-1β, SOD1: candidate biomarkers for PD progression | [10] |
| serum | PD (41), Ctl(41) | Caspase1: candidate biomarkers for PD αsyn | [3, 11] |

(NA ,not available; PD , Parkinson disease ;Ctl, control; ncRNA, noncoding RNA)

Reference

1. Zeng R, Luo DX, Li HP, Zhang QS, Lei SS, Chen JH: **MicroRNA-135b alleviates MPP(+)-mediated Parkinson's disease in in vitro model through suppressing FoxO1-induced NLRP3 inflammasome and pyroptosis**. *J Clin Neurosci* 2019, **65**:125-133.

2. Lv K, Liu Y, Zheng Y, Dai S, Yin P, Miao H: **Long non-coding RNA MALAT1 regulates cell proliferation and apoptosis via miR-135b-5p/GPNMB axis in Parkinson's disease cell model**. *Biol Res* 2021, **54**(1):10.

3. **Leaf-nosed bat**. In: *Encyclopædia Britannica.* Encyclopædia Britannica Online; 2009.

4. Nguyen TPN, Kumar M, Fedele E, Bonanno G, Bonifacino T: **MicroRNA Alteration, Application as Biomarkers, and Therapeutic Approaches in Neurodegenerative Diseases**. *Int J Mol Sci* 2022, **23**(9).

5. Zhang Q, Huang XM, Liao JX, Dong YK, Zhu JL, He CC, Huang J, Tang YW, Wu D, Tian JY: **LncRNA HOTAIR Promotes Neuronal Damage Through Facilitating NLRP3 Mediated-Pyroptosis Activation in Parkinson's Disease via Regulation of miR-326/ELAVL1 Axis**. *Cell Mol Neurobiol* 2021, **41**(8):1773-1786.

6. Ravanidis S, Bougea A, Papagiannakis N, Koros C, Simitsi AM, Pachi I, Breza M, Stefanis L, Doxakis E: **Validation of differentially expressed brain-enriched microRNAs in the plasma of PD patients**. *Ann Clin Transl Neurol* 2020, **7**(9):1594-1607.

7. Zhang YJ, Zhu WK, Qi FY, Che FY: **CircHIPK3 promotes neuroinflammation through regulation of the miR-124-3p/STAT3/NLRP3 signaling pathway in Parkinson's disease**. *Adv Clin Exp Med* 2023, **32**(3):315-329.

8. Kim R, Kim HJ, Kim A, Jang M, Kim A, Kim Y, Yoo D, Im JH, Choi JH, Jeon B: **Peripheral blood inflammatory markers in early Parkinson's disease**. *J Clin Neurosci* 2018, **58**:30-33.

9. Baran A, Bulut M, Kaya MC, Demirpençe Ö, Sevim B, Akıl E, Varol S: **High-sensitivity C-reactive protein and high mobility group box-1 levels in Parkinson's disease**. *Neurol Sci* 2019, **40**(1):167-173.

10. Roy A, Banerjee R, Choudhury S, Chatterjee K, Mondal B, Dey S, Kumar H: **Novel inflammasome and oxidative modulators in Parkinson's disease: A prospective study**. *Neurosci Lett* 2022, **786**:136768.

11. Wijeyekoon RS, Kronenberg-Versteeg D, Scott KM, Hayat S, Kuan WL, Evans JR, Breen DP, Cummins G, Jones JL, Clatworthy MR *et al*: **Peripheral innate immune and bacterial signals relate to clinical heterogeneity in Parkinson's disease**. *Brain Behav Immun* 2020, **87**:473-488.
