# Supplementary material for: The pyroptosis mediated biomarker pattern: an emerging diagnostic approach for Parkinson’s disease
Source: Cell Mol Biol Lett. 2024 Jan 3;29:7. doi: 10.1186/s11658-023-00516-y (PMC10765853; doi:10.1186/s11658-023-00516-y)
Supplement: Supplementary file 6 — Additional file 6: Figure S2. Transfection validation of miRNAs. [file 11658_2023_516_MOESM6_ESM.docx]

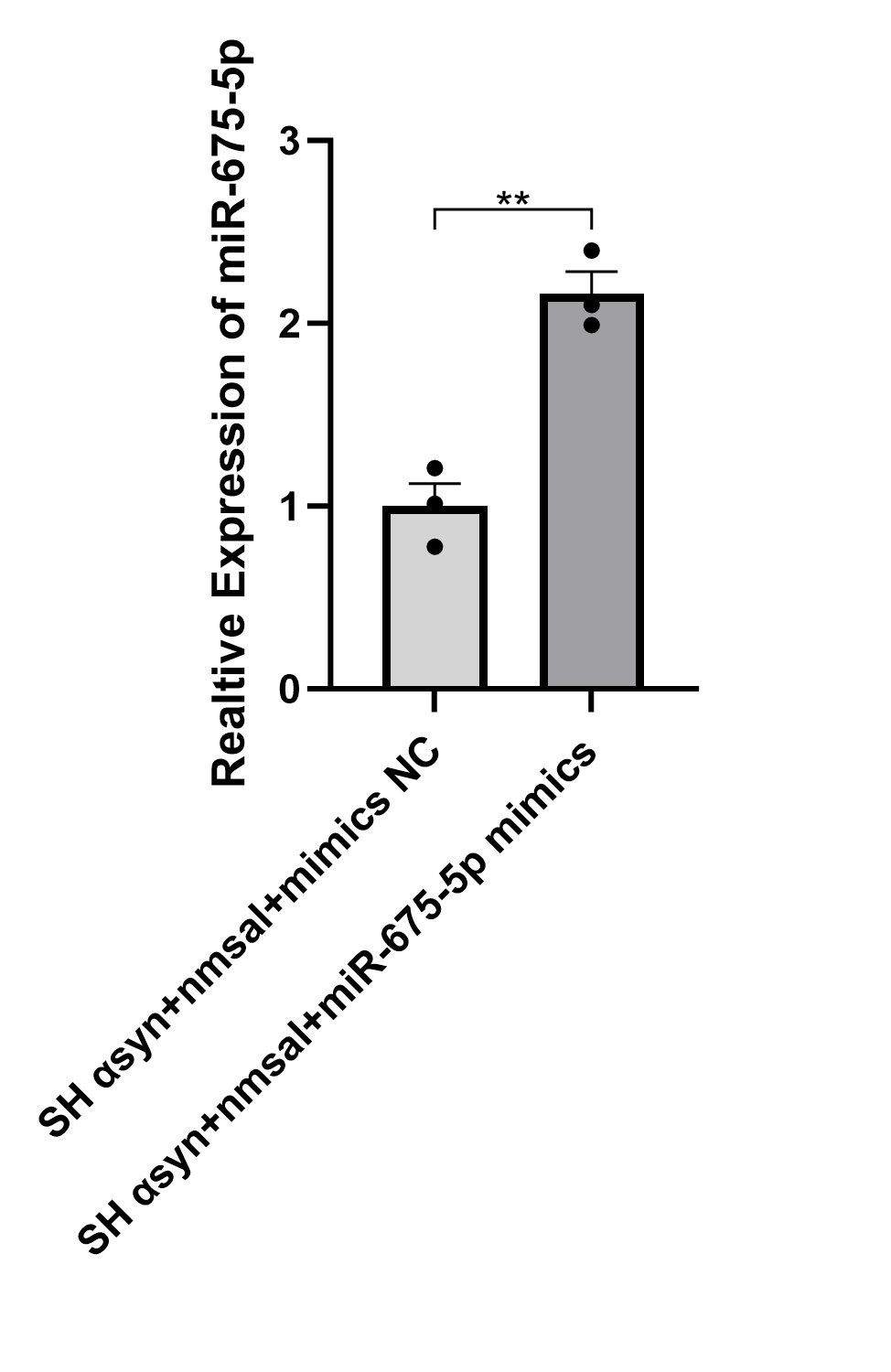

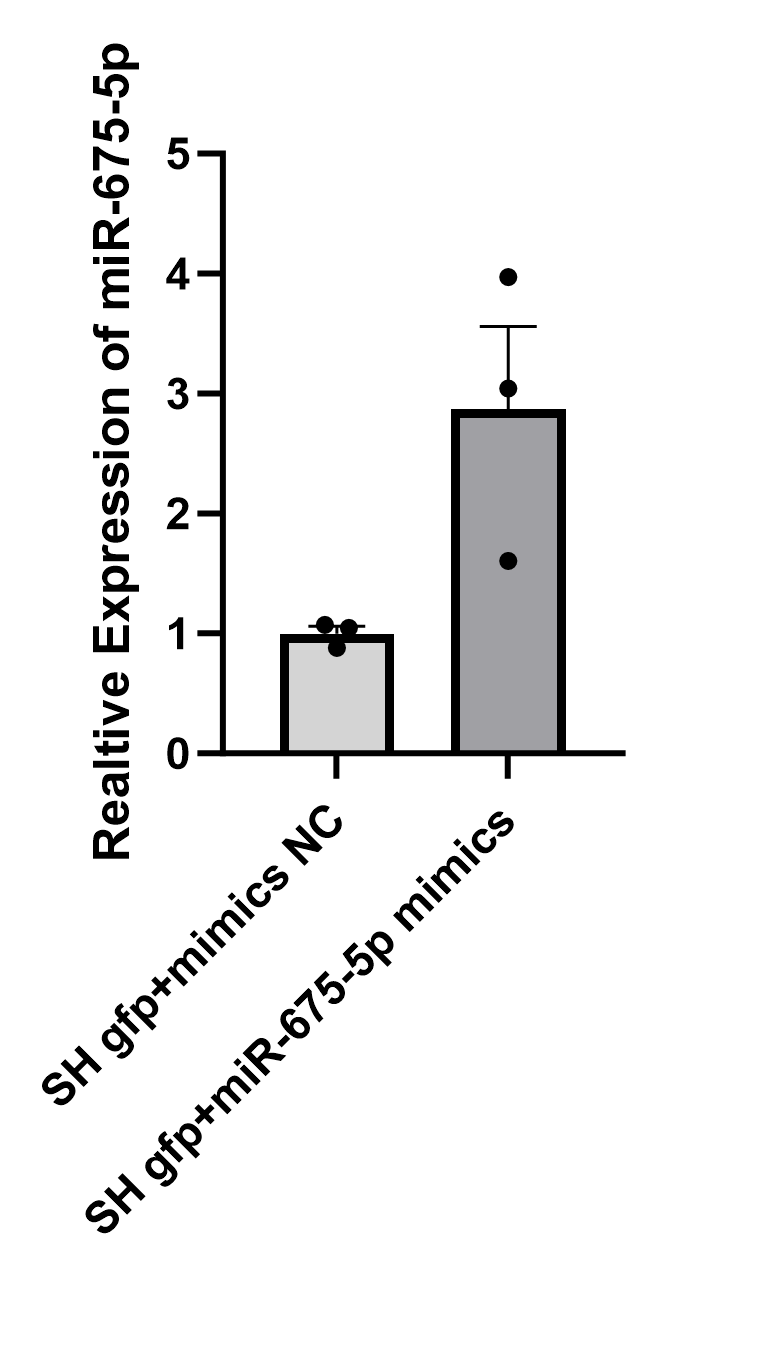
(A) (B)


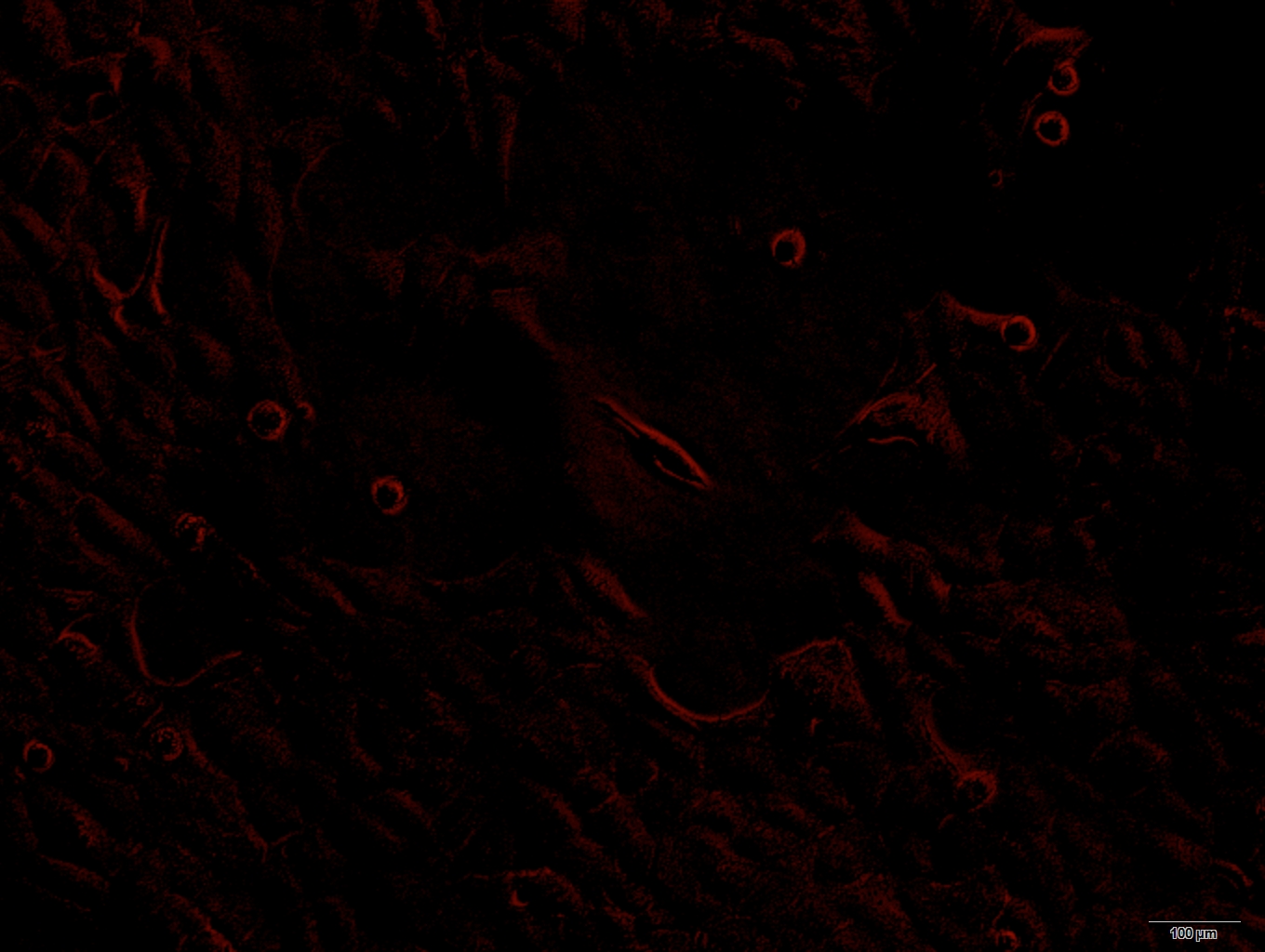

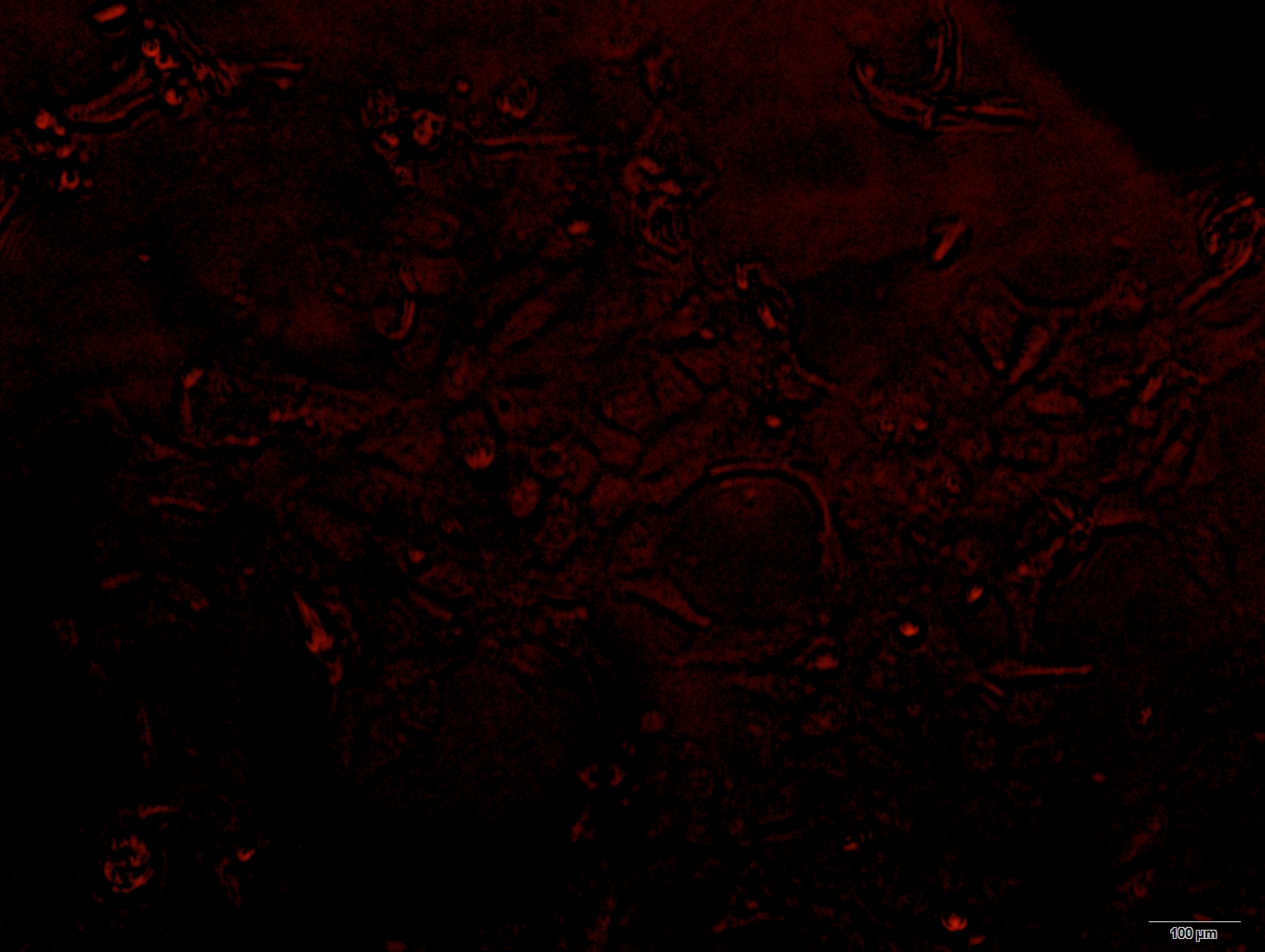
(C) (D)

Additional Fig 2 Transfection validation of miRNAs

The expression levels of miR-675-5p (A, B) were measured by RT-qPCR. The delta-delta cycle threshold value (2-ΔΔCt) method was used to analyze the results relative to U6. Data are represented as the mean ± SEM of three biological replicates, performed using three technical replicates. The transfections of miR-1247-5p inhibitor-Cy3 in SH gfp(C) and SH αsyn+ NM-Sal(D) were confirmed by Cytation3 imaging reader (BioTek, Vermont, VT, USA). (SH, SH-SY5Y cell; α syn, α-synuclein; NM-Sal, NM-Salsolinol; miR, microRNA; **P < 0.01)
